# Affinity-seq detects genome-wide PRDM9 binding sites and reveals the impact of prior chromatin modifications on mammalian recombination hotspot usage

**DOI:** 10.1186/s13072-015-0024-6

**Published:** 2015-09-07

**Authors:** Michael Walker, Timothy Billings, Christopher L. Baker, Natalie Powers, Hui Tian, Ruth L. Saxl, Kwangbom Choi, Matthew A. Hibbs, Gregory W. Carter, Mary Ann Handel, Kenneth Paigen, Petko M. Petkov

**Affiliations:** Center for Genome Dynamics, The Jackson Laboratory, 600 Main Street, Bar Harbor, ME 04609 USA

## Abstract

**Background:**

Genetic recombination plays an important role in evolution, facilitating the creation of new, favorable combinations of alleles and the removal of deleterious mutations by unlinking them from surrounding sequences. In most mammals, the placement of genetic crossovers is determined by the binding of PRDM9, a highly polymorphic protein with a long zinc finger array, to its cognate binding sites. It is one of over 800 genes encoding proteins with zinc finger domains in the human genome.

**Results:**

We report a novel technique, Affinity-seq, that for the first time identifies both the genome-wide binding sites of DNA-binding proteins and quantitates their relative affinities. We have applied this in vitro technique to PRDM9, the zinc-finger protein that activates genetic recombination, obtaining new information on the regulation of hotspots, whose locations and activities determine the recombination landscape. We identified 31,770 binding sites in the mouse genome for the PRDM9^Dom2^ variant. Comparing these results with hotspot usage in vivo, we find that less than half of potential PRDM9 binding sites are utilized in vivo. We show that hotspot usage is increased in actively transcribed genes and decreased in genomic regions containing H3K9me2/3 histone marks or bound to the nuclear lamina.

**Conclusions:**

These results show that a major factor determining whether a binding site will become an active hotspot and what its activity will be are constraints imposed by prior chromatin modifications on the ability of PRDM9 to bind to DNA in vivo. These constraints lead to the presence of long genomic regions depleted of recombination.

**Electronic supplementary material:**

The online version of this article (doi:10.1186/s13072-015-0024-6) contains supplementary material, which is available to authorized users.

## Background

Genetic recombination plays an important role in evolution, facilitating the creation of new, favorable combinations of alleles and the removal of deleterious mutations by unlinking them from surrounding sequences. Recombination also assures the proper segregation of homologous chromosomes at the first meiotic division, preventing aneuploidy. In mammals, as in yeast and higher plants, recombination is restricted to specialized sites along chromosomes, a kilobase or so in length, known as hotspots [1, 2], whose locations and relative activity determine patterns of inheritance from one generation to the next. There is now substantial evidence from population genetic studies of humans [3–5], genetic crosses in mice [6, 7] and cattle [8], and molecular studies of hotspots in mice [9–11], that recombination hotspot locations in mammals are determined by the zinc finger, DNA-binding protein PRDM9, which binds at recombination hotspots and trimethylates lysine 4 of histone H3 [10, 11].

PRDM9 is a highly polymorphic mammalian protein, with extensive variation reported both between and within species, including humans [3–7, 12], chimps [13, 14], cattle [8], equids [15], and mice, for which over 100 alleles have been reported [7, 16, 17]. The great majority of this variation occurs in the tandemly arrayed zinc-finger domain and involves changes in PRDM9’s DNA binding properties. Analyzing the binding properties of several individual PRDM9 binding sites in vitro, we previously found that binding requires the participation of every zinc finger in the PRDM9 tandem array, not only those that define the computationally derived binding motif, and that individual fingers vary in their contribution to determining binding specificity [18]. To extend these studies, we developed and now describe Affinity-seq as an efficient, generalized in vitro method for directly isolating and sequencing most genomic binding sites for a DNA binding protein and determining their relative binding affinities.

Despite the biological and evolutionary importance of mammalian hotspots, we lack an understanding of the factors and mechanisms that constrain their locations and activity. We have now used Affinity-seq to identify potential PRDM9 binding sites in vitro, and address the issue of identifying factors determining which of these sites are used in vivo and what their relative activity will be. We provide evidence that a set of prior chromatin modifications influences the likelihood that a potential PRDM9 binding site will be used in vivo. PRDM9 binding sites located in genomic regions with elevated levels of histone 3 lysine 9 di- or trimethylation (H3K9me2/3) or that are typically associated with the nuclear membrane protein Lamin B1 have a decreased likelihood of becoming activated, as measured by their acquisition of H3K4me3 or double-stranded breaks. Conversely, binding sites in protein-coding genes are more likely to become activated, and this effect increases in genes with higher levels of transcriptional activity in germ cells. The magnitude of these influences on binding site usage in vivo is inversely related to the relative binding affinity of PRDM9 in vitro. We conclude that the choice of which binding sites are used in vivo and what their relative activities will be reflects a balance between the intrinsic affinity of PRDM9 for its binding sites and the restrictions imposed by prior chromatin modifications. This association was further confirmed by ChIP-seq measurements of PRDM9 binding in vivo.

## Results

### Affinity-seq identifies genome-wide PRDM9 binding sites

The general principle of the Affinity-seq method is outlined in Additional file 1: Figure S1. A DNA-binding domain is cloned and tagged with 6His-HALO and then expressed in *E. coli*; the 6His tag facilitates protein purification (Additional file 2: Figure S2A), and the HALO tag contains a binding pocket that covalently binds ligands, facilitating attachment of a biotin moiety to the purified protein. DNA sheared to 180–200 bp is provided in considerable excess to provide competition between DNA binding sites. Following binding, DNA–protein complexes are then isolated on streptavidin beads and the DNA extracted for deep sequencing.

In the case of PRDM9, we used the terminal zinc finger domain of PRDM9^Dom2^ (PRDM9ΔZnF1^Dom2^, 412–847 aa), the allele present in C57BL/6J (B6) mice, which exhibits allele-specific binding of known hotspot sequences in vitro (Additional file 2: Figure S2B, hotspot specificity is as determined in [18]). This construct lacks the first zinc finger, which is widely separated from the remaining tandem array of 12 zinc fingers. Unlike the remaining fingers, which are highly polymorphic, this finger has been tightly conserved during mammalian evolution, suggesting an important regulatory role in vivo without being involved in determining DNA binding specificity. Expressed PRDM9^Dom2^ containing this separated finger (PRDM9ZnF1^Dom2^, 384–847 aa) has the same binding specificity; however, the efficiency of binding is severely reduced after purification (Additional file 2: Figure S2B). Its binding activity is partially restored when incubated with various other proteins in their native or denatured states (Additional file 2: Figure S2C) suggesting the importance of protein concentration in maintaining stability of purified PRDM9 and affecting its DNA binding activity (Additional file 2: Figure S2B).

Expressed PRDM9ΔZnF1^Dom2^ protein was partially purified by chromatography, first by SP-Sepharose and then on Ni^2+^ resin, to over 50 % purity estimated by silver staining (Additional file 2: Figure S2A). The purified protein is soluble and retains its DNA binding specificity as evidenced by its ability to bind synthetic oligonucleotides in vitro [18] (Additional file 2: Figure S2B) for up to 6 weeks at 4 °C without significant loss of activity. Further purification results in rapid loss of DNA binding activity and the protein cannot be stabilized by subsequent addition of extra protein (e.g. BSA). For this reason, a final purification step of PRDM9-DNA bound complexes is achieved in the Affinity-seq protocol itself (Additional file 2: Figure S2A).

**Fig. 1:**
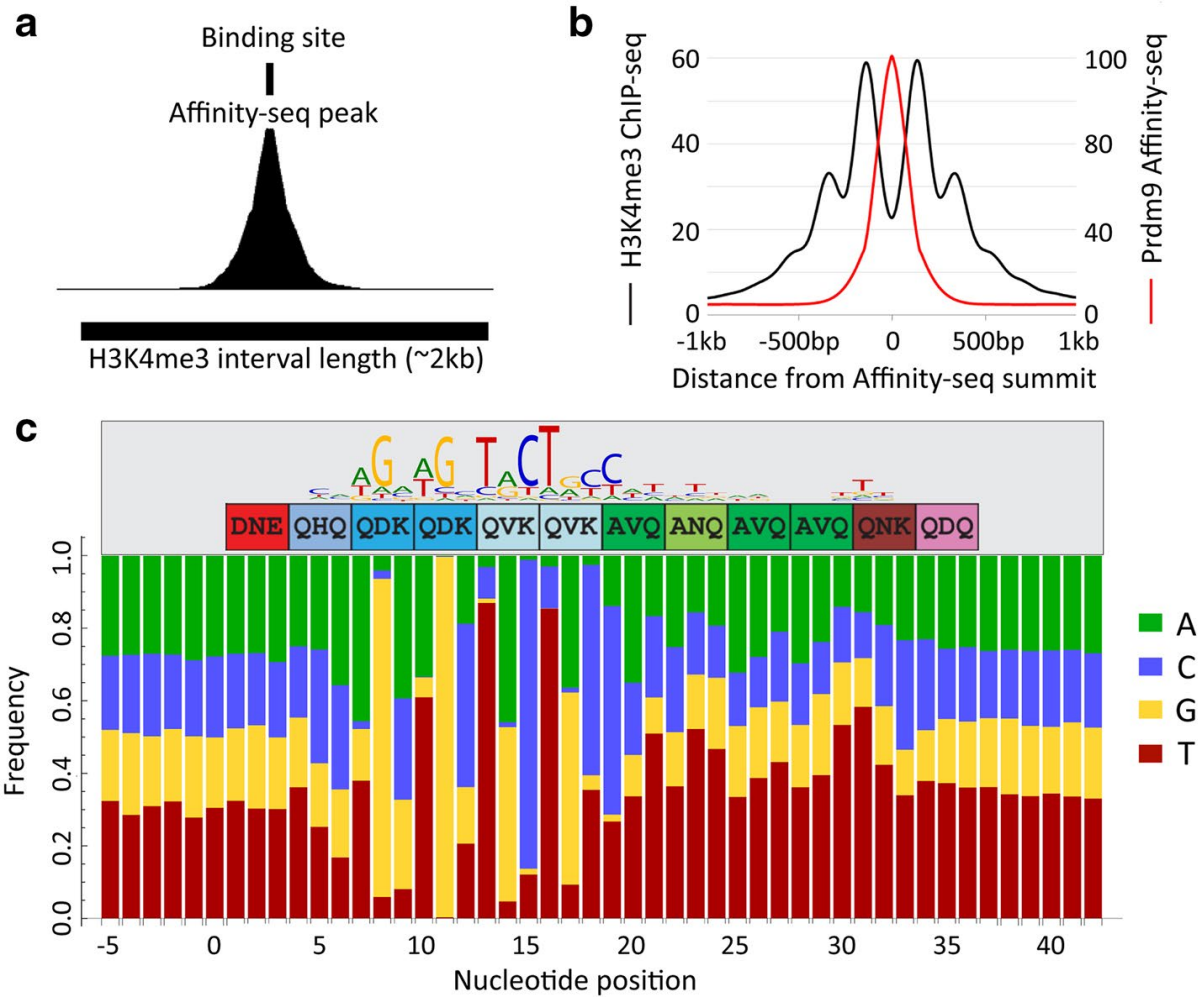
Affinity-seq determines genome-wide PRDM9 binding sites. **a** Shape of a representative Affinity-seq peak. The size and position of the inferred PRDM9 binding site are shown on top; the size and position of the corresponding H3K4me3 hotspot are shown on the bottom. **b** Comparison between the composite signals (obtained by aggregating across all hotspots in the genome) at H3K4me3 and Affinity-seq peaks centered on the inferred PRDM9 binding sites. **c** Distribution of nucleotide frequencies along PRDM9 binding sites including the flanking nucleotides. Distinct preferences can be seen over the entire binding sites; the strongest signals are detected at positions covered by the PRDM9^Dom2^ motif indicated on top (nucleotides 7–19). The sequence of the binding motif identified in the nucleosome-depleted regions of H3K4me3 peaks and the structure of the zinc finger domain of PRDM9^Dom2^ are shown above. Note that, except for the terminal fingers, nucleotide frequencies are distinctly different from genome average for all positions, including those outside the region of the computationally derived motif; that identical fingers (QDK, QVK and especially AVQ) vary in nucleotide frequencies depending on their location in the array, and that nucleotide frequencies at the same amino acid position (notably Q in fingers 3 and 4 v. 5 and 6) are strongly influenced by the identity of adjoining amino acids

Initially, we found 25,472 and 35,436 binding peaks at *p* < 0.01 threshold in two independent Affinity-seq experiments with purified PRDM9^Dom2^ protein variant, using input genomic DNA as a control. Of these, 24,033 peaks were common (Additional file 3: Figure S3A), with a good correlation of activities between the two experiments (*r*^*2*^ = 0.933, Additional file 3: Figure S3B). To improve the signal-to-noise ratio, which influences the false positive rate, we combined the data from the replicate samples and re-ran the peak-calling algorithm, again using input DNA as control. We obtained 31,770 PRDM9^Dom2^ binding sites (11.34 sequence peaks/Mb) in the B6 genome excluding the Y-chromosome. The peaks typically spanned a distance of ~320 bp (319.5–322.1 bp in 95 % bootstrap confidence interval), close to twice the length of the average DNA fragments minus the 34 bp length of a PRDM9^Dom2^ binding site determined previously [18] (Fig. 1a). A single PRDM9^Dom2^ binding motif matching the motif identified in in vivo studies [9, 11] was present at the central 150 bp of 96 % of the 31,770 sites. In contrast, no detectable motif was found in the adjacent 150-bp regions at both sides of the central region. Additionally, genomic positions of the PRDM9 binding sites coincided with the central nucleosome-depleted region present in H3K4me3 hotspots used in vivo [11] (Fig. 1b). Collectively, these results confirm that Affinity-seq correctly identifies PRDM9 binding sites.

We did not find a significant correlation between the motif match and binding strength which suggested that the nucleotides present at the remaining positions can also influence binding strength. We further investigated this possibility by determining the nucleotide bias along the entire binding site and surrounding nucleotides. The distribution of nucleotides at any position outside the binding sites did not differ from genome average. However, there was a clear nucleotide bias at the positions corresponding to the entire binding site, typically with a preference for one or two nucleotides (Fig. 1c), with the exception of the three nucleotides corresponding to the first finger and two nucleotides corresponding to the last zinc finger in the array. In five positions in the site (8, 11, 13, 15, and 16), the major nucleotides (8-G, 11-G, 13-T, 15-C, and 16-T) were present at >0.85 frequency and could be considered virtually invariable. The minimum length of the binding site for the 12-finger PRDM9^Dom2^ ZnF array is 36 bp, three bp per finger, if all fingers equally participate in DNA binding. The length of the nucleotide sequence differing from random nucleotide distribution (31 bp) is slightly shorter than the 36 bp needed to bind 12 zinc fingers and the 34 bp experimentally determined for the Pbx1 binding site [18] which suggests that the first and last fingers do not substantially contribute to sequence-specific binding.

### PRDM9 ChIP-seq

To provide comparison between in vitro and in vivo PRDM9 binding sites, we performed PRDM9 ChIP-seq in spermatocytes of 12–14 days post-partum (dpp) B6 mice, a time when germ cells are enriched for the pre-leptotene to zygotene stages of early meiosis and PRDM9 is active [11, 19], using a custom-made antibody [19]. We detected 1709 peaks, 1578 of which overlapped Affinity-seq binding sites. Although the PRDM9 ChIP-seq is much less sensitive than other methods for hotspot detection [19] and we recovered much lower number of PRDM9 ChIP-seq peaks, 92 % of them matched Affinity-seq binding sites, with overlapping peak centers (Additional file 4: Figure S4). Individual review of each of the 8 % additional peaks revealed a combination of false positives from the PRDM9 ChIP-seq and false negatives from the Affinity-seq assays.

### In vivo hotspots

Having a catalog of in vitro PRDM9 binding sites provided a new means of comparing in vitro and in vivo usage. In doing so we relied on the widely accepted model of recombination initiation in mammals, which supposes that hotspots are defined by the presence of a PRDM9 binding site where PRDM9 attaches and trimethylates H3K4me3, and that this arrangement determines the eventual site of double strand breaks (DSB) formation. PRDM9-dependent H3K4me3 marks first appear in early leptonema and persist until early pachynema, when they disappear with the advance of DNA repair process [20]. DSB formation follows similar dynamics as determined by DMC1 foci [21]. These stages can be assayed in vivo by ChIP-seq for their respective molecular tags: PRDM9, H3K4me3, and the single-stranded DNA-binding protein DMC1 that marks DSBs. These assays all have different sensitivities of detection; for this reason we consider that an in vivo hotspot exists in principle when one of the three in vivo measures coincides with an Affinity-seq binding site. ChIP-seq for H3K4me3 was carried out in spermatocytes from 12 to 14 dpp B6 males. The previously reported DMC1 data [9] was obtained using adult males. Because the H3K4me3 and DSB data are appreciably more sensitive and include all the PRDM9 ChIP-seq peaks, they together provided the means for a comprehensive analysis of in vivo hotspots. We found a total of 15,884 Affinity-seq binding sites overlapping either an H3K4me3 peak or a DMC1 peak. About 600 peaks were coincident between H3K4me3 peaks from both B6-Prdm9^Cst–KI^ and B6 [11] and had no DMC1 peak. These peaks were classified as ambiguous and removed leaving 15,244 sites to be designated as in vivo and 15,886 sites to be designated as in vitro only PRDM9^Dom2^ binding sites.

Using this characterization of hotspots, only about a half (48.0 %) of the 31,770 Affinity-seq binding sites are used in vivo to any measurable extent. However, the sites used in vivo did not have any significant feature differences from those detected in vitro only—the two groups of sites had same shape and width, and similar frequency of motif detection at the peak (97 % of in vivo and 93 % of in vitro only binding sites). No new motifs were detected in the remaining sites in either group. The nucleotide frequencies along the binding sites were indistinguishable between the two groups. (Additional file 5: Figure S5).

### Historical usage of Affinity-seq binding sites

**Fig. 2:**
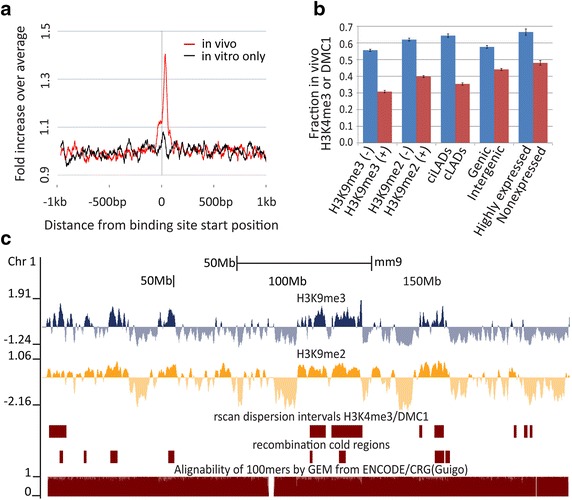
Hotspot usage and chromatin marks. **a** Detection of historical hotspot usage using CAST/EiJ genome for SNP calling. SNPs from the CAST/EiJ genome were obtained from the Wellcome Trust Sanger Institute’s Mouse Genomes Project. SNP densities are significantly increased at the PRDM9 binding sites of in vivo hotspots indicative of biased gene conversion at active hotspots, but only slightly so for sites that were not detectably active in vivo. Regions spanning 2 kb are centered and oriented based on the PRDM9 binding sites identified by Affinity-seq; SNP frequency for each nucleotide position is expressed as ratio of increase over the regional average. **b** Influence of genomic features on hotspot usage. The boundaries of H3K9me3 (+) or (−) and H3K9me2 (+) or (−) regions were determined using the corresponding ChIP-seq data and rseg. cLADs represent constitutive Lamin B-associated domains, ciLADS represent regions never associated with Lamin B domains. Gene expression is determined by ChIP-seq in 12-dpp spermatocytes. The *bars* show the fraction of in vivo used Affinity-seq binding sites relative to the total number of Affinity-seq binding sites in each category. The sites in each group are counted as belonging to a category if they cross into or are encapsulated within its borders. The fractions for various genomic features representative of *opened* and *closed* chromatin environments reveal a consistent reduction of in vivo usage in heterochromatic regions. Highly expressed genes represent the top quartile of the genes expressed in testes of 12-dpp mice. The *bars* show the fraction of in vivo used Affinity-seq binding sites relative to the total number of Affinity-seq binding sites in each category. *Standard error bars* are calculated from the Poisson distribution. **c** Distribution of H3K9me2, H3K9me3, and hotspot-deficient regions along mouse Chromosome 1. *Top to bottom* in each chromosome, *first panel*, H3K9me3 enriched (*up*) or depleted (*down*) regions; *second panel*, H3K9me2 enriched and depleted regions; *third panel*, regions containing Affinity-seq but deficient in H3K4me3/DMC1 peaks (in vivo Affinity-seq sites) in dispersed regions identified by Rscan are represented as *red rectangles*, those associated with assembly gaps removed; *fourth panel*, recombination-deficient regions in Collaborative Cross mice [30], *red rectangles*; *fifth panel*, alignability track from the UCSC browser showing assembly gaps and low mappability regions

DNA resected at the site of DSBs is repaired using the partner chromatid as the template, leading to the prediction that there should be strong evolutionary selection for mutations diminishing or inactivating the PRDM9 binding site [22]. This prediction has been confirmed by comparison of human hotspot sequences in chimpanzees [12], and experimentally by transferring a *Prdm9* allele from the sub-species in which it arose into a naive genetic background [19]. We found strong evidence that Affinity-seq binding sites detected as hotspots in vivo have accumulated mutations when their sequences were compared to the CAST/EiJ genome from the sub-species *Mus musculus castaneus* which lacks the PRDM9^Dom2^ variant and where PRDM9^Dom2^ hotspots were not used historically (Fig. 2a, red line) [19]. There is slight but not significant increase in mutation frequency at Affinity-seq binding sites not detected as hotspots in vivo, suggesting that a large proportion of these sites either have never been used or have been used so infrequently that they have undergone little evolutionary selection for mutations (Fig. 2a, black line).

### Heterochromatic features block hotspot activation

Under the conditions of Affinity-seq, all DNA sequences are equally available to PRDM9 binding; in contrast, DNA in vivo is organized in chromatin, raising the question whether chromatin imposed constraints on DNA access can influence binding site usage in vivo. To do so, we compared binding site usage in vivo in genomic regions enriched for H3K9me2 and H3K9me3, both known for their role in heterochromatin formation, with regions lacking these modifications. Large organized chromatin K9-modifications domains (LOCKs) represent closed chromatin regions characterized by H3K9me2 that vary in size and position in different cell types [23]. Likewise, H3K9me3 domains exhibit broad enrichment patterns that are associated with constitutive closed chromatin, but are also found in unique silenced loci [24]. The genome-wide extent of these two types of domains in germ cells from 12-dpp B6 males was determined by applying rseg, a Hidden Markov Model approach [25], to ChIP-seq data. Only 31 % of Affinity-seq binding sites were activated within H3K9me3 domains and 40 % within LOCKs/H3K9me2 (Fig. 2b), suggesting that prior chromatin modifications influence hotspot usage. In spermatocytes, as reported for other tissues [21, 22], these two types of marks cover broad domains along the chromosomes instead of forming distinct local peaks (Fig. 2c, Additional file 6: Figure S6, the two upper panels). The greater reduction in hotspot densities in H3K9me3 domains was associated with gene clusters whose expression is silenced in spermatocytes, including vomeronasal receptors and cytochrome P450s (Additional file 7: Figure S7). This reduction probably combines the inhibiting effects of closed chromatin and low gene expression levels (Fig. 2b, see below).

Considering the role of subnuclear localization of DNA in accessibility, we examined DNA regions that have been reported to be bound to the inner nuclear membrane protein Lamin B1 (cLADs) across a set of functionally unrelated mouse and human cell lines [26, 27]. We found that LADs reported in ES cell lines overlap strikingly with germ-cell LOCKs regions (Additional file 8: Figure S8) determined from our data. Similar overlap has been found in differentiated cell lines compared to ES cell lines [23]. Not surprisingly, hotspots are underrepresented in cLADs compared to DNA regions that are consistently not associated with cLADs (ciLADs) (Fig. 2b).

### Hotspot usage and gene expression levels

H3K4me3 hotspots are more frequent in protein-coding genes than in intergenic regions (Fig. 2b), confirming prior observations regarding DSB hotspots [10]. To test whether gene expression can affect hotspot usage, we performed RNA-seq in B6 12-dpp germ cells, finding a strong dependence of levels of hotspot densities on gene expression (Fig. 2b).

### Affinity-seq binding sites and hotspot distribution along the chromosomes

To obtain an independent measure of the distribution of hotspots along the chromosomes in vivo, we searched for genomic regions that are enriched or deficient in their content of either Affinity-seq binding sites or in vivo hotspots using Rscan statistics [28]. We found many hotspot-deficient regions lacking both H3K4me3 and DMC1 peaks, mostly overlapping with H3K9me2/3 domains (Fig. 2c, Additional file 6: Figure S6, third panel in each chromosome), but unlike our previous report of a torrid zone with multiple hotspots [29], we failed to find regions enriched for hotspots.

About 74 % of hotspot-deficient regions, spanning a total of 426.6 Mb, were not deficient in Affinity-seq binding sites compared to the genome-wide average, indicating that they are truly hotspot-deficient and not simply regions in which it is difficult to correctly assign sequencing reads. The hotspot-deficient regions are also deficient in genetic crossing over. Notable in this regard is the 13-Mb region on the X chromosome (113–126 Mb) (http://www.ncbi.nlm.nih.gov/geo/, GSE52628 [11]). Affinity-seq identified 122 binding sites, or 9.38 sites/Mb, in this region, only marginally lower than the genome-wide average of 11.34 peaks/Mb, showing that PRDM9 has no difficulty binding DNA in this region. Indeed, only 1.3 Mb of the entire 13-Mb region is reported as having mapping difficulties. Nevertheless, only 22 H3K4me3 or DMC1 hotspots were observed in this region, or 18 % of the detectable Affinity-seq binding sites compared to 48 % genome-wide. Moreover, this region is markedly deficient in genetic crossing over. Compared to the genome-wide average recombination rate of 0.67 cM/Mb in female mice, the genetic recombination rate across this region proved to be only 0.029 cM/Mb among 1092 offspring of female B6 X CAST/EiJ F1 hybrids carrying two different *Prdm9* alleles (*Prdm9*^*Dom2/Cst*^), and 0.023 cM/Mb among 912 offspring of NOD/LtJ X C3H/HeJ F1 hybrids carrying yet another *Prdm9* allele (*Prdm9*^*Dom3/Dom3*^) (Table 1), approximately 4 % of the genome-wide average. Similarly, 51 long regions having no crossovers (0.58–12.2 Mb) were found in all mouse chromosomes (except Chr 10 and Chr 11) in Collaborative Cross mice [30], where four different *Prdm9* alleles are segregating (Fig. 2c, Additional file 6: Figure S6, fourth panel in each chromosome). Sixty-nine percent or 35 of these regions, match the deficient regions reported here. All of them contain H3K9me2/3 enriched domains in spermatocytes and nuclear lamina domains from ES cells. Caution must be exercised when studying peak deficiency in these regions due to their repetitive nature even though their special chromatin environments make them particularly interesting. Remarkably, Liu et al. [30] discovered a high degree of overlap with segmental duplications occupying 47.7 % of cold regions.

**Table 1:** Recombination rates in the X-chromosome hotspot deficient region in female backcrosses

Cross	Interval	Interval size (Mb)	Number of recombinants	Number of progeny	Recombination rate (cM/Mb)
C57BL/6J × CAST/EiJ	rs3653678–rs3670542	15.96	5	1092	0.029
NOD/LtJ × C3H/HeJ	rs3653678–rs3656160	14.08	3	912	0.023

The special value of the genetic data is that it describes recombination deficiency within these regions without being susceptible to any of the pitfalls of massive parallel sequencing alignments. Taken together with the molecular data, it appears that many of the genetically deficient regions derive from the failure of PRDM9 to activate recombination there, and that the deficient regions are common to multiple *Prdm9* alleles due to their closed chromatin state.

### DNA binding and hotspot usage

**Fig. 3:**
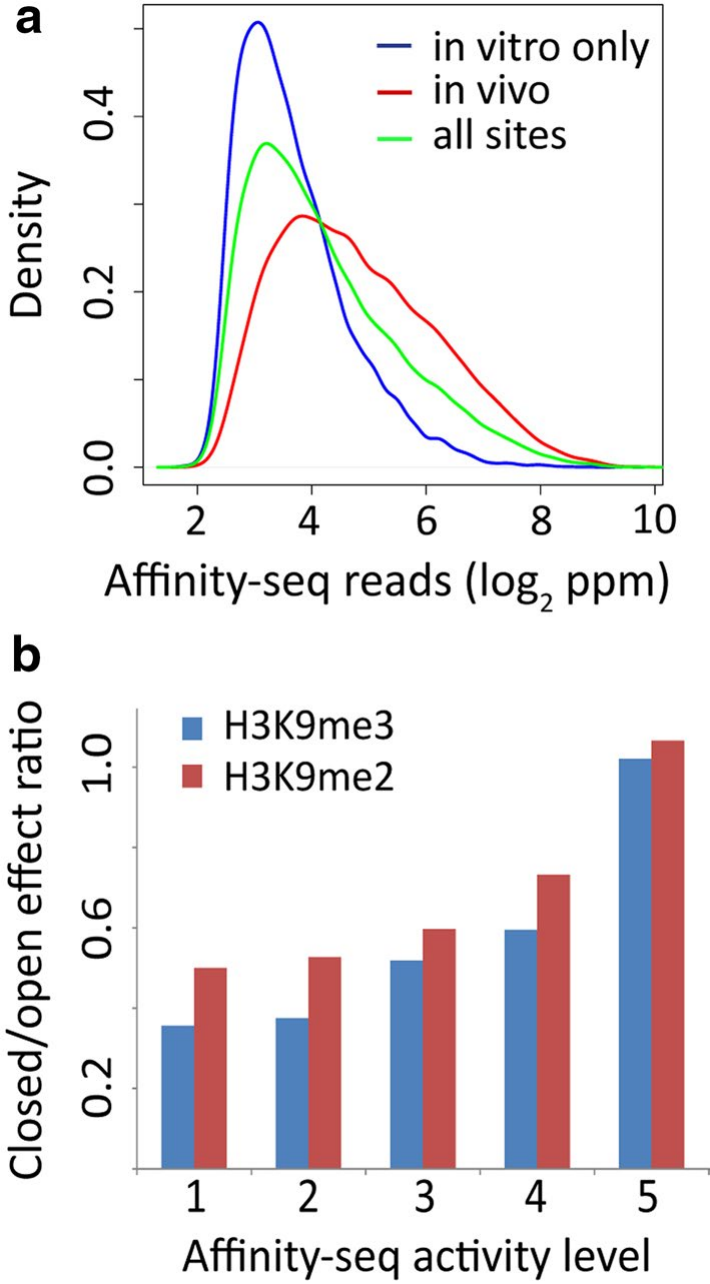
Affinity-seq binding site activities measured in vitro influence hotspot detection in vivo. **a** PRDM9 affinity to DNA and chromatin features determine hotspot activation qualitatively and quantitatively. Density distribution of Affinity-seq binding sites in hotspots detected in vivo (*blue*) or not detected (in vitro only, *red*) in spermatocytes compared to all sites (*green*). Active hotspots contain Affinity-seq binding sites with higher average activity compared to the sites detected in vitro only. **b** Closed chromatin state defined by H3K9me2/3 marks impacts lower affinity hotspots more strongly than higher affinity hotspots. We ranked hotspots by their affinity for PRDM9 determined by Affinity-seq and divided them into five equal quintiles. For each group, we then calculated the ratio of hotspot number to total number of Affinity-seq sites in regions of closed chromatin (H3K9me2 or 3 positive) to regions of open chromatin (lacking H3K9me2 or me3). A ratio of 1 indicates no effect of heterochromatin. These data indicate that the strength of binding can increasingly overcome the effects of heterochromatin

Figure 3a shows that among the many binding sites with similar PRDM9 affinities, only some are activated in vivo, indicating that activation involves an additional local factor. Figure 3b shows that this local factor depends on prior chromatin modifications and is overcome at sites with higher, intrinsic PRDM9 binding affinities (level 5 on Fig. 3b). In vitro, under equilibrium conditions the affinity of PRDM9 binding to a DNA site, *K*_d_, is the ratio of the *off* rate to the *on* rate of attachment. For macromolecular reactions, *on* rates are typically diffusion-limited, with the consequence that differences in *K*_d_ between hotspots describe differences in the *off* rate, which is inversely proportional to the average residence time of PRDM9 on its binding site.

**Fig. 4:**
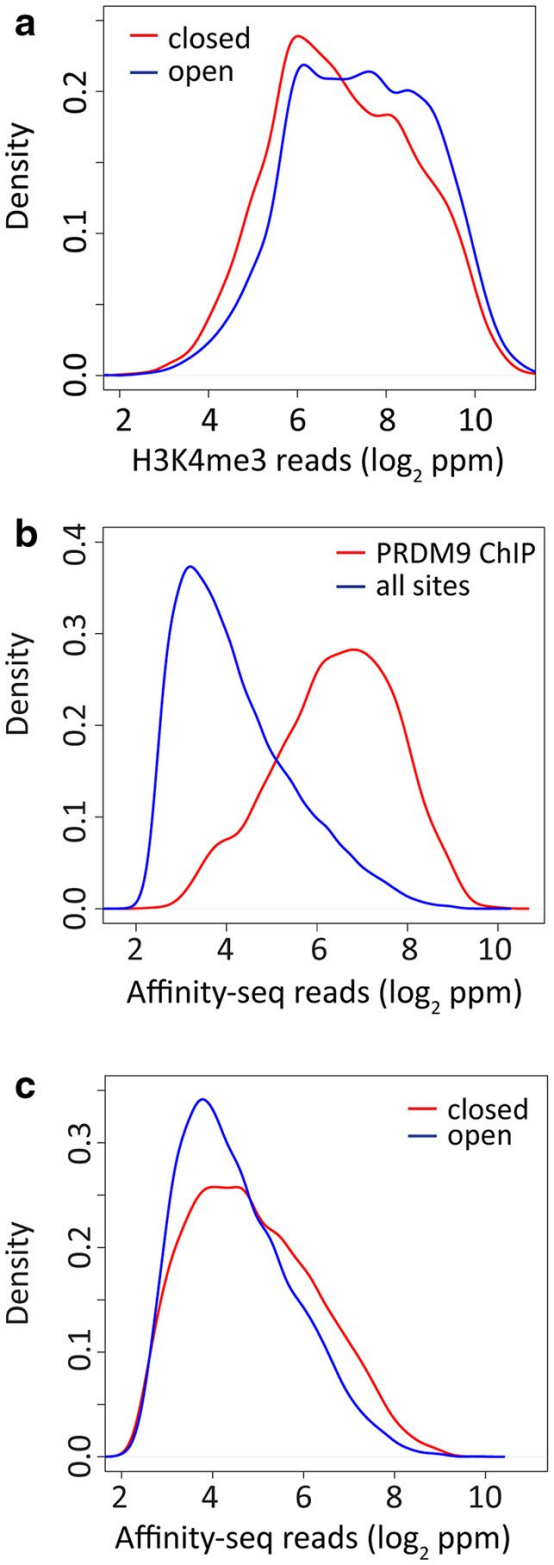
Affinity-seq and H3K4me3 peak strength in closed vs. open chromatin. **a** Density plots of H3K4me3 signals in closed versus open chromatin as determined by the absence or presence of H3K9me2/3 enrichment reveal that H3K4me3 signals are stronger at hotspots in open chromatin (*blue*) than in closed chromatin (*red*). In this analysis, DMC1 hotspots overlapping promoters were excluded. In all *three panels*, in order to eliminate effects of filtering multi-mapped reads only peaks which span regions of 100 % uniqueness in mappability were selected. Mann–Whitney test gives a *p* value <2.2 × 10^−16^ that distributions are the same. **b** PRDM9 ChIP peaks are found at the sites with strongest affinity to DNA in vitro. Density plots are shown for Affinity-seq read counts normalized to parts per million at peaks for those sites detected by PRDM9 ChIP assay (*red*) and all sites (*blue*). Mann–Whitney test gives a *p* value <2.2 × 10^−16^ that distributions are the same. **c** Stronger affinities to DNA are detected at PRDM9 ChIP-seq peaks found in closed compared to open chromatin. Density plots are shown for Affinity-seq read counts normalized to parts per million at peaks in H3K9me2/3 enriched regions (*red*) and peaks at regions not enriched for H3K9me2/3 (*blue*). Mann–Whitney test gives a *p* value <2.2 × 10^−16^ that distributions are the same

In spermatocytes, the local chromatin state can create an energy barrier to PRDM9 binding in vivo. When this barrier is greater than the intrinsic energy of binding at a hotspot, binding is completely suppressed, and in regions such as closed chromatin where this barrier is typically higher, the result is a reduction in hotspot density. At hotspots where the barrier is only partial, it has the effect of reducing the net energy of binding, increasing the effective *K*_d_ in vivo, and reducing the residence time of PRDM9. If the residence time determines the likelihood that PRDM9 will remain long enough to successfully trimethylate a hotspot, we would expect H3K4me3 levels at hotspots in closed chromatin to be typically less than at hotspots in open chromatin, and this is indeed the case (Fig. 4a).

Similar reasoning explains the observations that the PRDM9 ChIP sites we find in vivo are typically those with the highest affinities in vitro (Fig. 4b). Since we find only the top 10 % of all in vivo hotspots by PRDM9 ChIP (1578 out of the 15,244 detected by H3K4me3 and/or DMC1 hotspots), it is reasonable to assume that the more strongly PRDM9 molecules binds to DNA, the longer it will remain bound, increasing the chance of detecting interaction by ChIP. The difference between closed and open chromatin hotspots in the distribution of their in vitro PRDM9 affinities can be explained along the same lines—the energy barrier to binding is higher in heterochromatic regions, with the consequence that we detect hotspots with higher average DNA binding affinity there (Fig. 4c).

Overall, we conclude that whether PRDM9 binding sites are used in vivo, and what their relative activities will be when they are used, depends on a local balance between the affinity of PRDM9 for its binding site and an energy barrier created by local chromatin modifications, and that these effects can have a major influence on genetic recombination patterns within species.

## Discussion

### Affinity-seq provides a means for genome-wide determination of long zinc finger protein binding sites

The human genome contains over 800 genes encoding proteins with zinc finger domains [31], more than half of which contain eight or more fingers organized in a tandem fashion. Many of these genes function as transcription factors, insulator binding proteins, or chromatin modifiers. Previous methods of genome-wide analysis for examining DNA–protein interactions [32–34] have employed multiple rounds of selection and/or randomized oligonucleotide DNA targets, approaches that either fail to determine relative binding affinities directly or to determine the actual repertoire of genomic binding sites. In contrast, Affinity-seq directly determines the relative affinity of tens of thousands of binding sites genome-wide with high binding specificity but without the complications of successive rounds of selection. It also provides the opportunity for mutational analysis of binding site specificities using alternate sources of genomic DNA. Using this assay, we showed that PRDM9 binding specificity depends entirely on the terminal, tandem array of zinc fingers; the presence or absence of the first, widely separated, zinc finger may affect solubility and/or stability but not the binding specificity. Although it is possible that adjacent or more remote protein domains might still affect binding specificity in other proteins, our results suggest that isolated ZnF domains can correctly represent the binding specificity of the entire protein in vivo.

In evidence of Affinity-seq’s utility, the present data clearly indicate that the binding specificity of the PRDM9 zinc finger domain depends on all of the fingers except the first and last, not just those determining the extracted binding motif. Moreover, fingers with the same structure have different binding specificities depending on their location in the array (Fig. 1c). We expect that the Affinity-seq strategy will prove applicable to other zinc-finger proteins and likely to other classes of DNA-binding proteins as well, providing a significant technology for exploring the molecular bases of DNA binding specificity beyond simply finding motifs.

Among Affinity-seq binding sites, about a half of them corresponds in location to biologically active recombination hotspots. They are precisely located at the central nucleosome deficient region characteristic of hotspots (Fig. 1b) and have the same computationally derived consensus binding site (Fig. 1c) as that derived from hotspots defined by sites of H3K4 methylation [11] or DMC1 binding [9]. Multiple lines of evidence suggest that the other half of Affinity-seq binding sites, those not detected as biological hotspots in vivo, are also true PRDM9 binding sites whose in vivo ability to activate recombination is suppressed by other biological factors. The two sets of sites, those detected in vivo and those not, have the same consensus PRDM9 binding motif derived by MEME and virtually the same frequency distribution of bases at each position in the binding site (Additional file 5: Figure S5). No previously undetected motifs were found in either group. What differences there are between the two groups, are best explained by the bias created by prior chromatin modifications towards stronger affinities for the sites used in vivo.

Given these results, it is reasonable to conclude that Affinity-seq correctly identifies intrinsic PRDM9 binding sites in genomic DNAs and that the observed constraints on binding site usage in vivo correctly identify relevant biological factors.

### Chromatin structure and hotspot usage

Early indications that differences in chromatin structure might play a role in hotspot usage in non-PRDM9 organisms came with findings that hotspots in the budding yeast *S. cerevisiae* occur at nuclease-hypersensitive sites [35, 36]; that yeast recombination hotspots are marked by H3K4me3, and loss of the H3K4 methyltransferase SET1 results in the disappearance of normal hotspots with the emergence of new ones [37]; and that loss of the histone deacetylase SIR2 alters the distribution of yeast hotspots [38]. In contrast, in the fission yeast *S. pombe*, the role of H3K4me3 is replaced by acetylated H3K9 [39], a histone modification that is similarly considered as opening chromatin. The first indication that prior chromatin modifications might influence mammalian hotspot activation came with the finding by Smagulova et al. [10] that mammalian hotspots are more likely to occur in genic rather than intergenic regions. Among mammalian chromatin modifications that are likely to be involved, we now show that H3K9me2/3 marks are associated with a barrier to PRDM9 binding. Conversely, histone modifications that accompany transcription are associated with a lower threshold for PRDM9 binding to meiotic DNA as we find higher hotspot usage in gene bodies of expressed genes (Fig. 2b). These modifications include faster turnover of histone acetylation marks and H3K36me3 [40–42] and the acquisition of ubiquitinated H2BK120 [43, 44]. H3K36me3, like H3K4me3, is a mark of chromatin activation, and H2BK120ubi prevents compaction of chromatin into higher order structures [45]. Heterochromatin-specific and gene expression-specific marks probably affect hotspot usage independently. Silenced genes acquire tissue-specific H3K9me3 marks (Additional file 7: Figure S7) making it harder for PRDM9 to bind there. During transcription, the gene expression associated H3K36me3 mark replaces H3K36ac, stabilizes the nucleosomes at the gene bodies and prevents cryptic transcription [46, 47], possibly facilitating PRDM9 binding and DSB initiation as well. Interestingly, PRDM9 has recently been shown to trimethylate H3K36 in addition to H3K4 in vitro [48], suggesting a possible role of this histone modification in recombination initiation.

## Conclusions

Our work provides a novel approach for studying DNA–protein interactions. Its application to PRDM9 leads to the finding that recombination hotspot usage is modulated by the local chromatin state, which can reasonably be explained by the latter’s influence on the energetics of PRDM9 binding. These constraints lead to the presence of long genomic regions depleted of recombination. The approach described here provides a conceptual framework for the search for additional factors controlling hotspot usage.

## Methods

### Cloning of 6His-HALO-Prdm9

The C-terminal fragment of PRDM9^Dom2^ lacking the separate zinc finger (412–843 AA) was cloned into pH6HTN His6HaloTag^®^ T7 Vector (Promega) using Pvu I—Not I restriction sites.

### Expression and cell lysate preparation

The protein was expressed in Rosetta cells (Merck Millipore) at 37 °C for 4 h. The cells were centrifuged at 3000*g* for 5 min, and the cell pellet from 100 ml culture was resuspended in 8 ml of 50 mM sodium phosphate, 100 mM sodium chloride, pH 8.0, containing 16 mg lysozyme, and incubated on ice 30’. The lysate was frozen overnight at −80 °C. The next day, the lysate was sonicated using three 10-s bursts at medium intensity, then frozen and sonicated again under the same conditions.

The first purification step—ion exchange on SP-Sepharose (cat.#17-1087-01, GE Healthcare Life Sciences, Piscataway, NJ, USA), was performed using an NaCl step-wise gradient. The active protein was eluted at 300 mM salt concentration. The active fractions were pooled and subjected to a second round of purification on affinity chromatography using Ni^2+^ resin (cat.#88221, Thermo Scientific Pierce, Rockford, IL, USA) according to manufacturer’s specifications. Active fractions were eluted at 250 mM imidazole.

The quantity of the purified 6His-HALO-PRDM9 was determined by band comparison to BSA standards on silver-stained gels.

### Affinity-seq

Genomic DNA was sonicated to an average of ~200 bp length using a Covaris M220 high-speed digital sonicating water bath (Covaris, Inc., Woburn, MA, USA). In a separate tube, HaloTag^®^ PEG-Biotin Ligand (cat. #G8591, Promega Corporation, Madison, WI, USA) was added to 100 µl purified 6His-HALO-PRDM9 (~2.5 pM) to 1 µM final concentration and the mixture was incubated 2–4 h at room temperature.

Binding reaction was performed by combining 20 μl of 10 × binding buffer (100 mM Tris, 500 mM KCl, 10 mM DTT pH 7.5), 10 μl of 1 % NP40, 20 µg sonicated DNA (3–5 × 10^12^ binding sites) and 7 µl of PEG-Biotin labeled protein sample (~0.16 pM protein or ~10^11^ molecules). The reaction mixture was incubated for 4 h at room temperature with rotation.

Fifty microliters MyOne T1 streptavidin beads (cat. #65601, Life Technologies Thermo Fisher Scientific) were washed three times with 0.5 ml TBST (TBS containing 0.05 % Tween 20), capturing particles with a magnet between washes. After the last wash, the bound samples were added and incubated for 30 min at room temperature on a rotating wheel. The beads were washed three times with 1.0 ml TBST containing 0.5 mg/ml BSA.

Protein-DNA complexes were eluted from the beads by adding 24 µl 1 % SDS and 16 μl 20 mM Tris pH 7.5, and incubating at 68 °C overnight with shaking. DNA was purified through Qiagen mini-elute (cat #28206, Qiagen) column. The eluted DNA was used to prepare an Illumina^®^ sequencing library using the ChIP-seq protocol. About 80 million reads were obtained from each individual library.

### DNA sequence analysis

Two replicate Affinity-seq samples were sequenced at 100-bp reads using the Illumina HiSeq 2500 and subsequently trimmed for quality using trimmomatic. Alignments to the mm9 mouse genome were obtained utilizing BWA v1.2.3 [49] with default parameters and reads which failed to align to unique positions in the genome were discarded. This achieved alignments of 68.89 million reads in one sample and 105.53 million reads in another. Peaks were called individually with MACS2 at a *p* value threshold of 0.01 utilizing a control dataset obtained by sequencing the input DNA and subsequently compared. Both datasets were subjected individually to motif analysis with MEME Suite (v 4.10.1) for motif discovery and sequence searching [50] using default parameters. For each site, a *p* value threshold of 0.001 was used. The 150 bp central peak regions were processed by MEME on a chromosome by chromosome basis. In all runs only a single motif was ever found, which matched previously published B6 motifs with only subtle differences between nucleotide frequencies. The surrounding 150 bp regions on both sides of the peak regions were tested separately; no significant motif was found.

SNPs between C57BL/6J and CAST/EiJ were obtained from the Wellcome Trust Sanger Institute’s Mouse Genomes Project (http://www.sanger.ac.uk/resources/mouse/genomes/).

### H3K4me3 ChIP

ChIP-seq data for anti-H3K4me3 obtained from C57BL/6J (B6) and the co-isogenic strain, B6-Prdm9^CAST-KI/Kpgn^ (B6.P9^Cst^), which has the CAST/EiJ *Prdm9* allele placed into the C57Bl/6J background, were reported by Baker et al. [11]. Data are available at NCBI Gene Expression Omnibus (GEO; http://www.ncbi/nlm/nih.gov/geo/) under accession number GSE52628.

### H3K9me2 and H3K9me3 ChIP

ChIP for either of these two histone modifications was carried out using an identical protocol to that used for H3K4me3 ChIP, except using antibody to H3K9me2 (Abcam ab1220) or H3K9me3 (Active Motif 39766). The sequence files were subjected to the same pipeline utilized for the Affinity-seq samples.

For the H3K9me2/3 samples peaks were called using a Rseg, a Hidden Markov approach to determining long domains of enrichment over a control samples. Enriched regions were visualized by performing chromosome walks of both sample and control and calculating a log base twofold ratio for reads in every 2 kb interval.

### PRDM9 ChIP

This ChIP was performed using antibody against the N-terminal fragment of mouse PRDM9 (101–170 aa) elicited in guinea pig. The ChIP protocol and analysis were carried out as described in [19]. PRDM9 ChIP samples were subjected to the same pipeline utilized for the Affinity-seq samples. Peaks for PRDM9 ChIP were called using MACS1.4 at a *p* value threshold of 0.00001 and removing duplicate reads.

### RNA-seq

RNA was extracted from four replicates of B6 germ cells enriched from testes at 12 dpp by enzymatic digestion. For each sample, mRNA was extracted from total RNA, fragmented, and purified. Barcoded sequencing libraries were prepared according to manufacturer’s protocols (Illumina^®^; San Diego, CA, USA). Briefly, double-stranded cDNA was made using random primers, overhangs were converted into phosphorylated blunt ends, and sample-specific adaptors (including 8-bp “barcodes”) were ligated to the DNA fragments. PCR was performed to enrich for the adapter-modified DNA fragments, and the libraries were validated using an Agilent Technologies 2100 Bioanalyzer. Sequencing was performed by The Jackson Laboratory’s Gene Expression Service using the Illumina^®^ HiSeq platform. Each sample was sequenced to a depth of approximately 25 million 100-bp, paired-end reads.

The RNA-seq data underwent pre-processing and quality control measures, including initial data calibration and filtering using Illumina^®^ Inc.’s Real Time Analysis (RTA) software and chastity filter. Gene expression levels were quantified by aligning reads to the mouse reference genome (NCBI build 37) using the BowTie 0.12.9 alignment software [51], using the NCBIm37 transcriptome as a reference. We filtered out reads with two or more mismatches against the reference transcriptome, accepting those with the minimum number of mismatches for each of 100 bp read (‘–all’, ‘–best’, and ‘–strata’ options were used). Normalized expression level per gene was calculated in fragments per kilobase of transcript per million mapped reads (FPKM) using RSEM version 1.2.8 using the following parameters: –fragment–length–mean 280 and –fragment–length–sd 50 [52].

### Statistical analysis of DNA-seq and RNA-seq data

Custom software for processing bedgraph, BAM/SAM files and bedfiles, and RNA-Seq data were developed in the Java programming language containing open-source frameworks BioJava v3.04 and SAM-JDK version 1.92. Bioinformatics tools utilized in analyses included Illumina^®^ Casava v1.8, BWA v1.2.3 [49], samtools v0.1.19 based on SAM Spec v1.4 [53], and BEDTools v2.17.0 [54].

### Rscan

Regions deficient in H3K4me3/DMC1 in vivo hotspots along chromosomes were determined using an *r*-scan statistic based on the Karlin and Macken method in which the significance of spans is calculated empirically to take into account ends of chromosomes [28]. Rscans are distance measurements between subjects that are near one another but not always adjacent. Additional degrees of dispersion can be detected by looking at distances between near neighbors where gaps between adjacent members may not be significantly large yet collectively a group of consecutive subjects can be significantly distanced from one another.

Given a genome of *n*_c_ hotspots on each chromosome, c, there are *n*_c_ − 1 interhotspot intervals or distances that are the difference in rank between the *i*th and (*i* + 1)th hotspots, denoted as *U*_*i*_. For any chromosome with *n*_c_ hotspots, the base-pair distances in *r*-scan lengths (*r* = 1, 2, 3,…) between hotspots is calculated as follows:

$$R_{i}^{\left( r \right)} = \mathop \sum \limits_{j = 1}^{i + r + 1} Uj, \quad i = 1, 2, \ldots ,n_{\text{c}} - r$$

The set of *r*-scan lengths from all chromosomes, *R*_*i*_^(*r*)^, is ranked in descending order. Let *m*_*k*_^(*r*)^ be the *k*th largest *r*-scan length from the ordered set, *R*_*i*_^(*r*)^. For each observed *r*-scan length in the test set, the probability of its being due to chance was determined from the cumulative frequency distribution.

For any given set of genomic locations we evaluate algorithmically distance measurements between locations that range from immediately consecutive distances between locations to locations that are up to 24 consecutive distances apart from one another. Using 10,000 sets of randomly selected hotspots equal in number to the test set, the cumulative frequency distribution of $m_{k}^{(r)}$ for all *k* and 1 ≤ *r* ≤ 24 were computed. Only the top 10 ranked distances for each consecutive distance are evaluated. Probabilities assigned to *R*_*i*_^(*r*)^ observed in the test set were adjusted using the Benjamini–Hochberg false discovery rate method [55]. Test-set hotspots were denoted as dispersed if they were bounded by an *r*-scan length that was the 10th or lower ranked length (*k* ≤ 10) and had an adjusted probability less than 0.05. Significant *r*-scan intervals were merged together to form putative hotspot-deficient regions. An ascending ordered list is utilized for clustering analysis and a descending ordered list for dispersion analysis.

Effectively, this approach is looking for unusually long distances not only between adjacent hotspots, but also for accumulation of dispersion of distances over up to 24 interhotspot intervals, and allows merging adjacent dispersed intervals. Given the average interhotspot distance of 87 kb between Affinity-seq binding sites and 182 kb between the in vivo hotspots, the approach can detect hotspot-depleted regions on a Megabase scale size.

## Data access

Data have been deposited at NCBI’s Gene Expression Omnibus and are accessible through GEO Series accession number GSE61613 (http://www.ncbi.nlm.nih.gov/geo/query/acc.cgi?acc=GSE61613).

## Additional files

Additional file 1:
**Figure S1.** Outline of the Affinity-seq method. Additional file 2:
**Figure S2.** Fig. S2A Purification of expressed 6His-HALO-PRDM9ΔZnF1 for Affinity-seq. Fig. S2B. Variant-specific binding of expressed 6His-HALO-PRDM9^Dom2^ in vitro. Fig. S2C. Partial restoration of PRDM9ZnF1 DNA binding activity by protein addition. Additional file 3:
**Figure S3.** Comparison of Affinity-seq replicate samples. Additional file 4:
**Figure S4.** Comparison between the composite signals at H3K4me3 ChIP, PRDM9-ChIP and Affinity-seq binding sites centered on the inferred PRDM9 binding sites. Additional file 5:
**Figure S5.** Comparison of distributions of nucleotide frequencies along PRDM9 binding sites used in vivo and in vitro only including the flanking nucleotides. Additional file 6:
**Figure S6.** Closed chromatin marks, hotspot-deficient regions and recombination cold spots along individual mouse chromosomes. Additional file 7:
**Figure S7.** Hotspot densities are reduced in gene clusters with silenced expression in spermatocytes. Additional file 8:
**Figure S8.** Comparison between LOCKs (H3K9me2) in germ cells and LADs in ES cells.

## Abbreviations

ChIP-seq: chromatin immunoprecipitation followed by deep sequencing
ZnF: zinc finger
B6: C57BL/6J mouse inbred line
LOCKs: large organized chromatin K9-modifications domains
cLADs: constitutive lamin-associated domains
