# Supplementary figures and images for: Affinity-seq detects genome-wide PRDM9 binding sites and reveals the impact of prior chromatin modifications on mammalian recombination hotspot usage
Source: Epigenetics Chromatin. 2015 Sep 7;8:31. doi: 10.1186/s13072-015-0024-6 (PMC4562113; doi:10.1186/s13072-015-0024-6)

Additional file 1:

Figure S1. Outline of the Affinity-seq method.

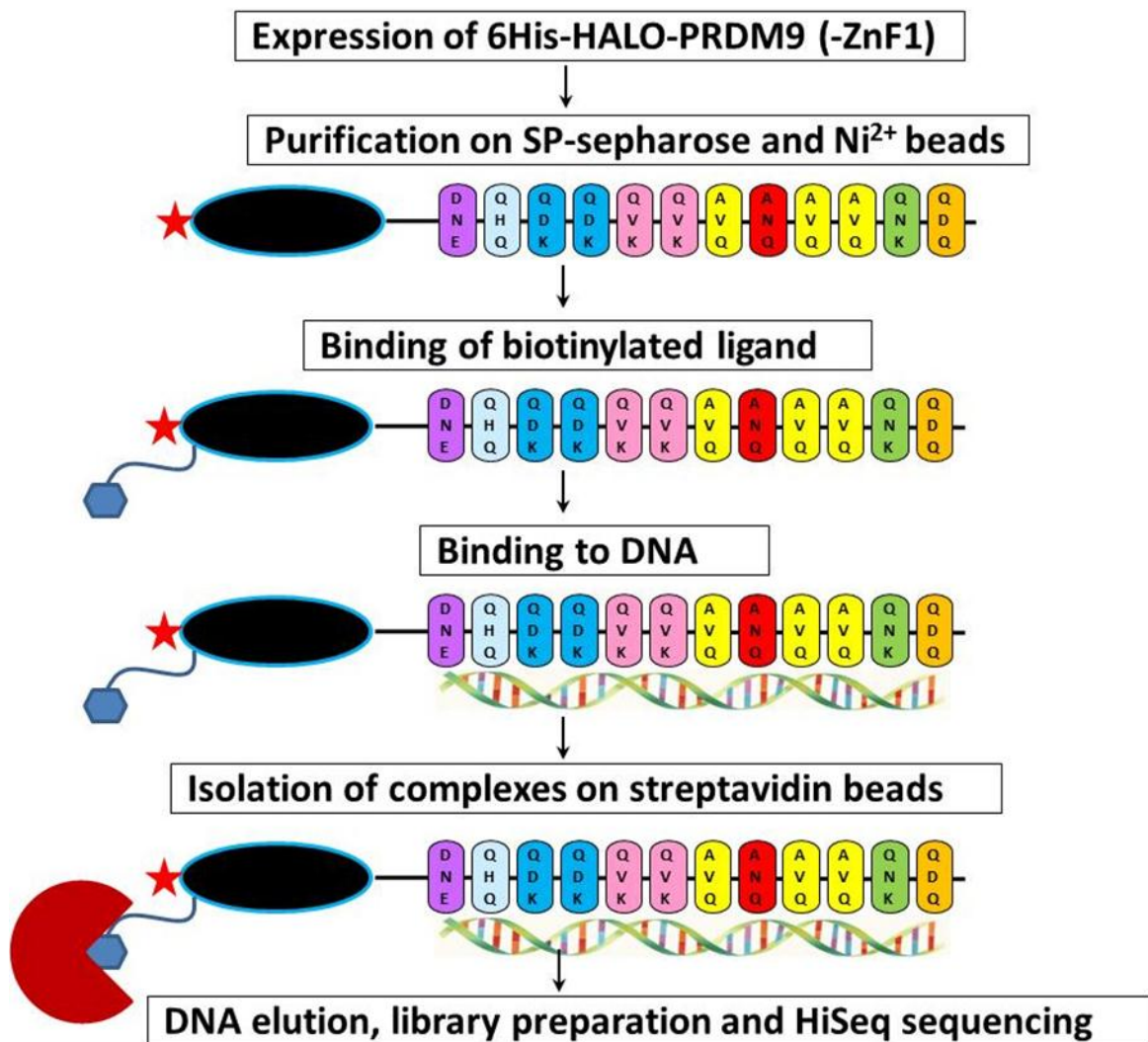

Supplement: Additional file 1: — Figure S1. Outline of the Affinity-seq method. [file 13072_2015_24_MOESM1_ESM.pdf]
