# Supplementary material for: Affinity-seq detects genome-wide PRDM9 binding sites and reveals the impact of prior chromatin modifications on mammalian recombination hotspot usage
Source: Epigenetics Chromatin. 2015 Sep 7;8:31. doi: 10.1186/s13072-015-0024-6 (PMC4562113; doi:10.1186/s13072-015-0024-6)

Additional file 2:

Figure S2A. **Purification of expressed 6His-HALO-PRDM9ΔZnF1 for Affinity-seq**.

a. Coomassie brilliant blue staining of purified 6His-HALO-PRDM9^{Dom2}ΔZnF1. Molecular weight markers are on the right. **b**. Western blot with antibodies against 6His tag. The band of interest is indicated by an arrow. Molecular weight markers are on the left. The sizes of molecular weigth markers are shown between the two panels. In each figure, Lane 1 – Combined fractions of 500 mM and 1000 mM NaCl after SP-sepharose; lane 2 – 250 mM imidazole fraction after affinity purification on Ni^{2+}-sepharose; 3 – eluate after purification on streptavidin beads.

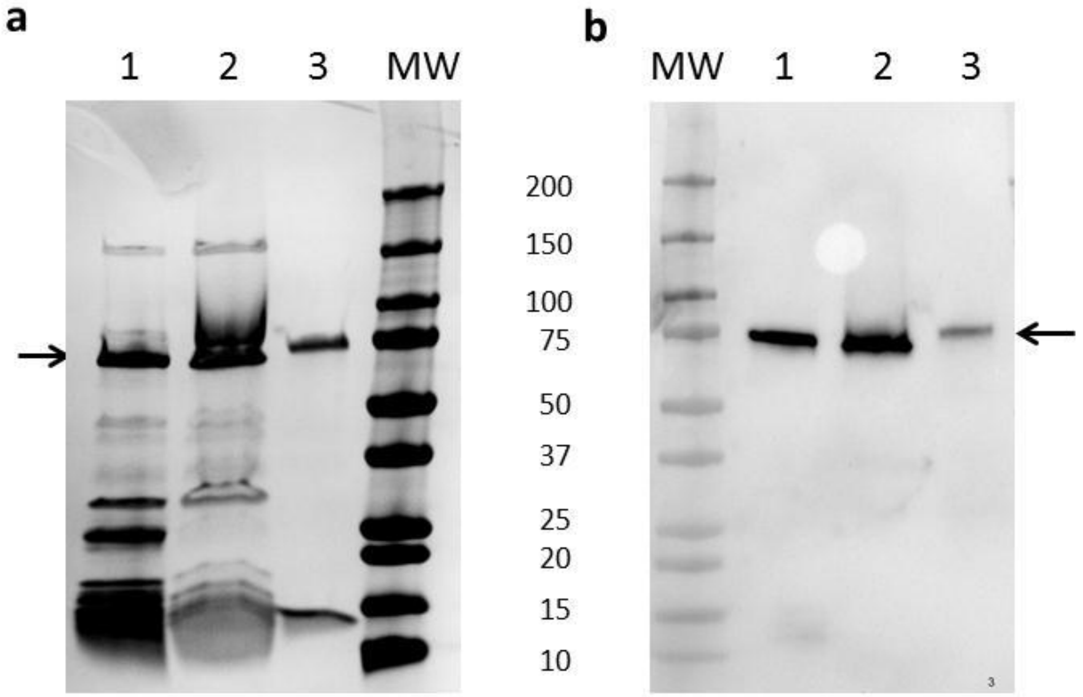

Fig. S2B. **Variant-specific binding of expressed 6His-HALO-PRDM9^{Dom2} *in vitro***. Expressed and purified 6His-HALO-PRDM9 C-terminal fragment lacking the separated zinc finger (PRDM9ΔZnF1) shows variant-specific binding. Hotspot *Pbx1* is activated by PRDM9^{Dom2} *in vivo*; hotspot *Hlx1* is activated by PRDM9^{Cst}. PRDM9^{Dom2}ΔZnF1 binds *Pbx1* (lanes 2-3) but not *Hlx1* (lanes 8-9). PRDM9^{Cst}ΔZnF1 binds *Hlx1* (lane 12) but not *Pbx1* (lane 6). Purified PRDM9ZnF1 (containing the separated zinc finger) shows nearly absent DNA binding activity (lane 4) but it is partially restored by adding mouse testis protein extract (lane 5). Binding specificity was tested by electrophoretic mobility shift assay (EMSA) as described in Billings et al., 2013 [18]. Biotin-labeled double-stranded oligo representing a variant-specific binding site was incubated with purified PRDM9 variant. Pbx1 oligo was added to lanes 1-6, Hlx1 oligo to lanes 7-12. Labeled oligo alone, black arrow. Shifted band (red arrow) indicates specific PRDM9-DNA binding. The composition of the reaction mixtures is shown above the figure.

	1	2	3	4	5	6	7	8	9	10	11	12
Pbx1	+	+	+	+	+	+	−	−	−	−	−	−
Hlx1	−	−	−	−	−	−	+	+	+	+	+	+
PRDM9ΔZnF1^{Dom2}	−	+	+	−	−	−	−	+	+	−	−	−
PRDM9ΔZnF1^{Cst}	−	−	−	−	−	+	−	−	−	−	−	+
PRDM9ZnF1^{Dom2}	−	−	−	+	+	−	−	−	−	+	+	−
Testis extract	−	−	+	−	+	−	−	−	+	−	+	−

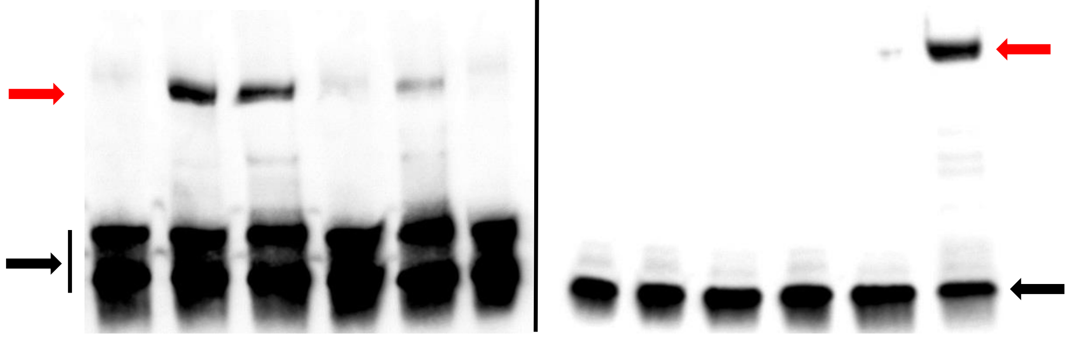

Fig. S2C. Partial restoration of PRDM9ZnF1 DNA binding activity by protein addition. Expressed and purified 6His-HALO-PRDM9 C-terminal fragment containing the separated zinc finger ($PRDM9ZnF1^{Dom2}$) shows nearly absent DNA binding activity as shown in Fig. S2B. It is partially restored by adding various non-related proteins in native or denatured state. Lane 1, Pbx1 labeled oligo (no protein); lane 2, Pbx1 + $PRDM9ZnF1^{Dom2}$; lanes 3-7 – as in 2, with addition of 1 mg/ml final concentration of the following proteins: 3 – mouse testis extract (MT), 4 – mouse testis extract denatured by heating to 100°C for 20' (MTD), 5 – bovine serum albumin (BSA), 6 – *E.coli* proteins (EC), 7 – mouse liver extract (ML). The position of the shifted band is indicated by red arrow on the right. Labeled oligo alone, black arrow. Binding specificity was tested by EMSA as in Fig.S2B.

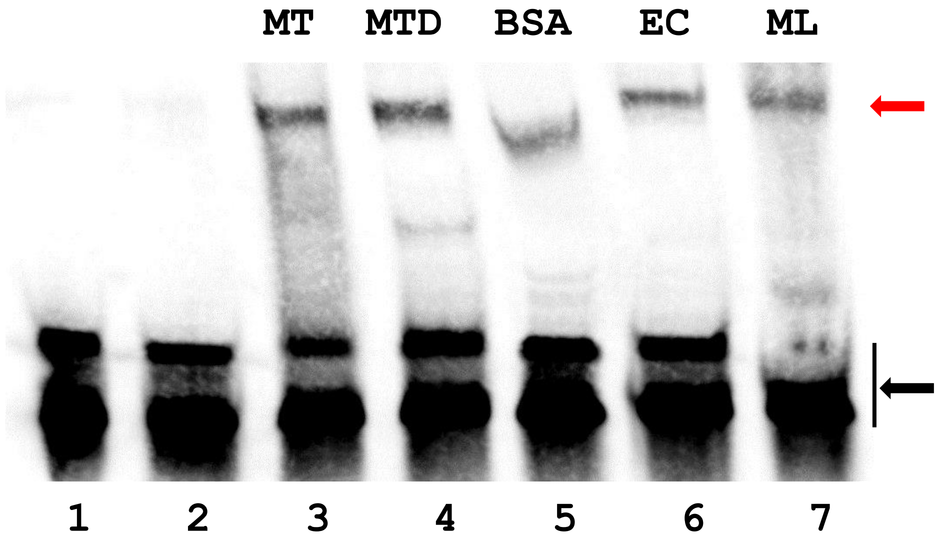

Supplement: Additional file 2: — Figure S2. Fig. S2A Purification of expressed 6His-HALO-PRDM9ΔZnF1 for Affinity-seq. Fig. S2B. Variant-specific binding of expressed 6His-HALO-PRDM9Dom2 in vitro. Fig. S2C. Partial restoration of PRDM9ZnF1 DNA binding activity by protein addition. [file 13072_2015_24_MOESM2_ESM.pdf]
