# Supplementary material for: Affinity-seq detects genome-wide PRDM9 binding sites and reveals the impact of prior chromatin modifications on mammalian recombination hotspot usage
Source: Epigenetics Chromatin. 2015 Sep 7;8:31. doi: 10.1186/s13072-015-0024-6 (PMC4562113; doi:10.1186/s13072-015-0024-6)

Additional file 3:

Figure S3. Comparison of Affinity-seq replicate samples.

A. Venn diagram showing the overlap of Affinity-seq binding sites in two separate experiments. Replicate 2 was sequenced at greater read depth than replicate 1.

B. Regression line of normalized read counts for common peaks between replicates.

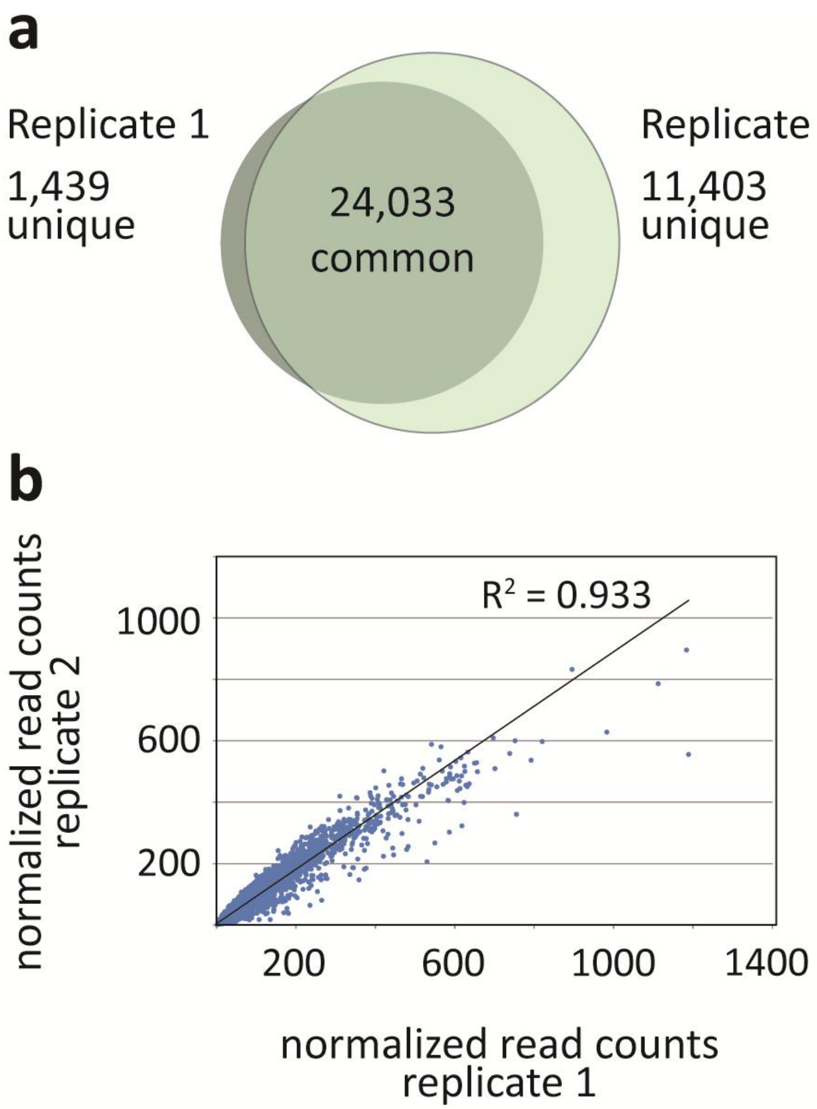

Supplement: Additional file 3: — Figure S3. Comparison of Affinity-seq replicate samples. [file 13072_2015_24_MOESM3_ESM.pdf]
