# Supplementary material for: Affinity-seq detects genome-wide PRDM9 binding sites and reveals the impact of prior chromatin modifications on mammalian recombination hotspot usage
Source: Epigenetics Chromatin. 2015 Sep 7;8:31. doi: 10.1186/s13072-015-0024-6 (PMC4562113; doi:10.1186/s13072-015-0024-6)

Additional file 4:

Figure S4. Comparison between the composite signals at H3K4me3 ChIP, PRDM9-ChIP and Affinity-seq binding sites centered on the inferred PRDM9 binding sites. Blue line, H3K4me3 ChIP sites; red line, Affinity-seq sites; black line, PRDM9 ChIP sites. There is a complete overlap between Affinity-seq and PRDM9 ChIP-seq composite peaks, which fall in the nucleosome-depleted region in the center of the H3K4me3 composite peak. The narrower size of the Affinity-seq binding sites compared to PRDM9 ChIP-seq binding sites is due to the shorter and more homogenous DNA fragment size achieved with naked DNA (Affininty-seq) rather than chromatin-bound DNA (ChIP-seq).

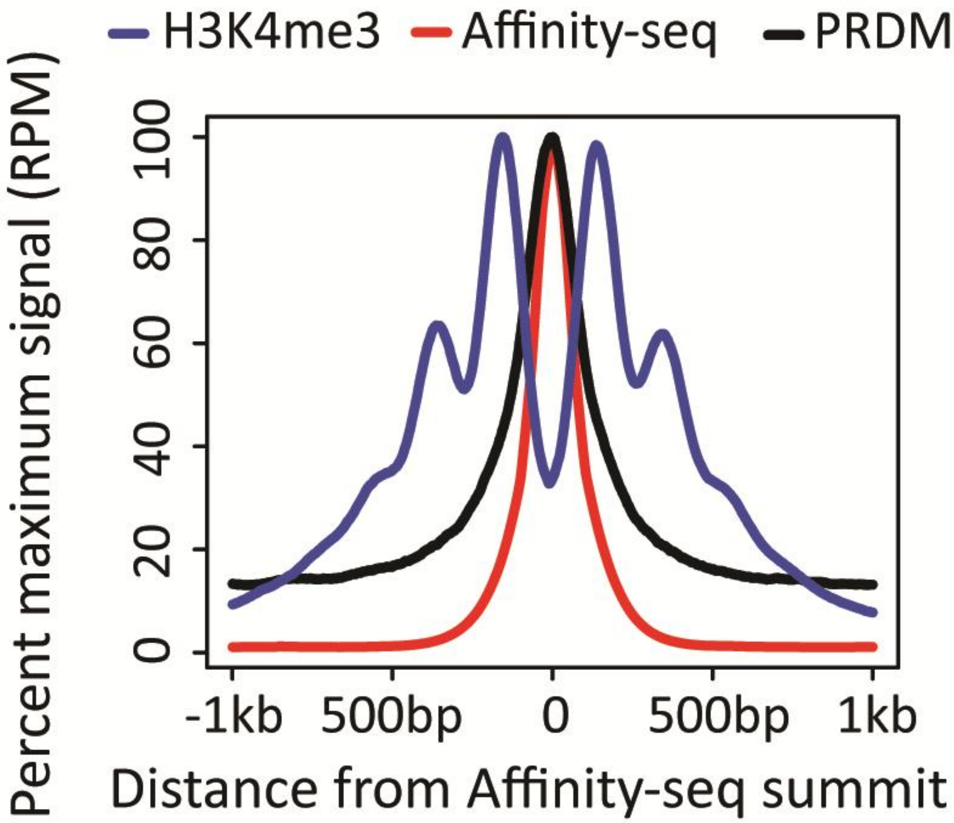

Supplement: Additional file 4: — Figure S4. Comparison between the composite signals at H3K4me3 ChIP, PRDM9-ChIP and Affinity-seq binding sites centered on the inferred PRDM9 binding sites. [file 13072_2015_24_MOESM4_ESM.pdf]
