# Supplementary material for: Affinity-seq detects genome-wide PRDM9 binding sites and reveals the impact of prior chromatin modifications on mammalian recombination hotspot usage
Source: Epigenetics Chromatin. 2015 Sep 7;8:31. doi: 10.1186/s13072-015-0024-6 (PMC4562113; doi:10.1186/s13072-015-0024-6)

Additional file 5:

Figure S5. Comparison of distributions of nucleotide frequencies along PRDM9 binding sites used *in vivo* and *in vitro* only including the flanking nucleotides. Top panel, Affinity-seq binding sites used *in vivo*; bottom panel, Affinity-seq binding sites detected *in vitro* only. No appreciable difference was found when comparing nucleotide frequency distributions of in vivo versus in vitro only Affinity-seq sites. All sites were oriented and centered based on the PRDM9 binding site as detected by MEME motif analysis. The regions span the 36 base pair binding site starting at position one and includes six flanking nucleotides on either side.

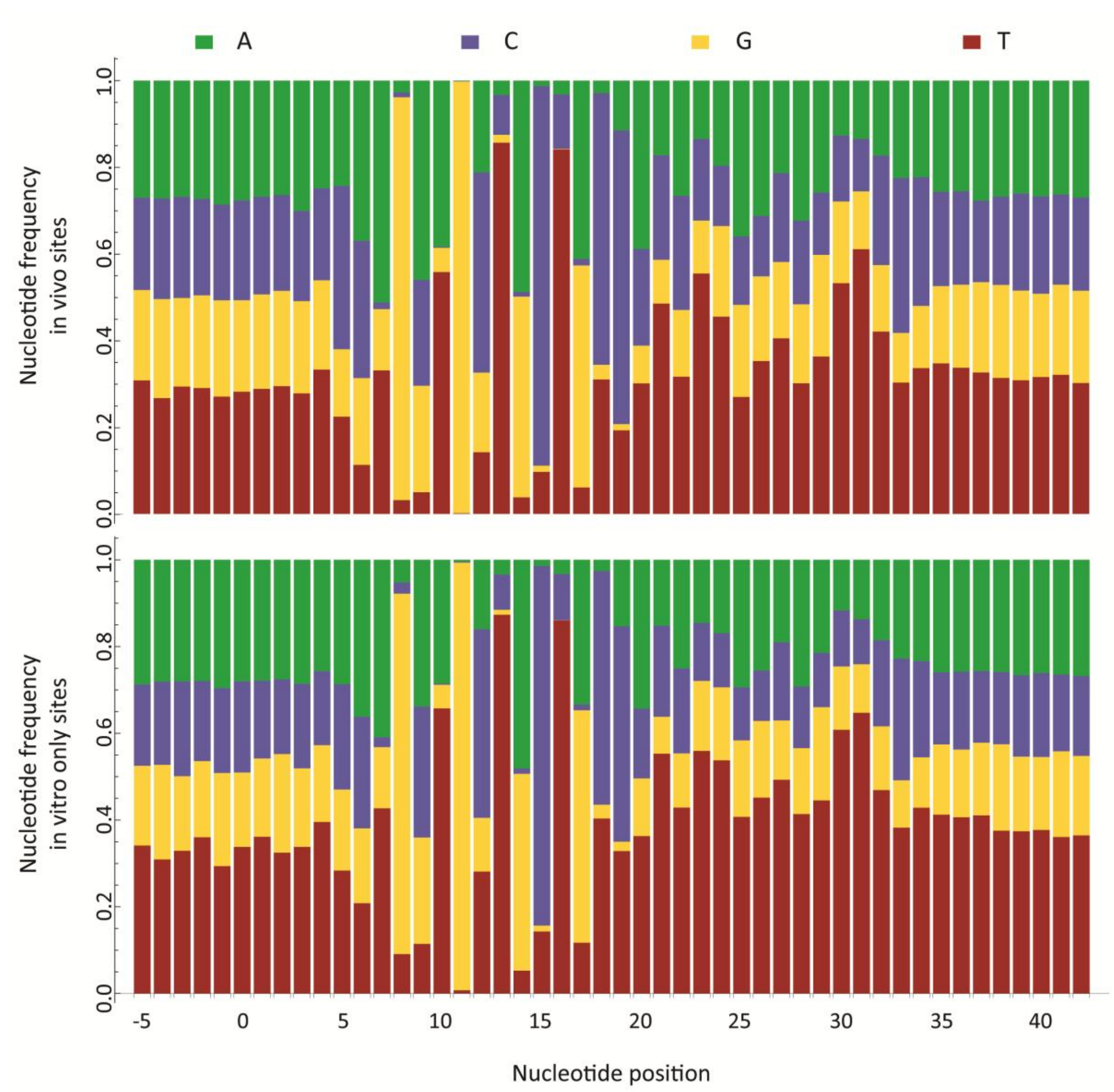

Supplement: Additional file 5: — Figure S5. Comparison of distributions of nucleotide frequencies along PRDM9 binding sites used in vivo and in vitro only including the flanking nucleotides. [file 13072_2015_24_MOESM5_ESM.pdf]
