# Supplementary material for: Affinity-seq detects genome-wide PRDM9 binding sites and reveals the impact of prior chromatin modifications on mammalian recombination hotspot usage
Source: Epigenetics Chromatin. 2015 Sep 7;8:31. doi: 10.1186/s13072-015-0024-6 (PMC4562113; doi:10.1186/s13072-015-0024-6)
Supplement: Additional file 6: — Figure S6. Closed chromatin marks, hotspot-deficient regions and recombination cold spots along individual mouse chromosomes. [file 13072_2015_24_MOESM6_ESM.pdf]

Additional file 6:

Figure S6. Closed chromatin marks, hotspot-deficient regions and recombination cold spots along individual mouse chromosomes. Top to bottom in each chromosome – H3K9me3 enriched (up) or depleted (down) regions; H3K9me2 enriched and depleted regions; regions containing Affinity-seq *in vivo* binding sites in dispersed regions identified by Rscan are represented as red rectangles; regions containing recombination cold spots in Collaborative Cross mice [32] – red rectangles; fifth panel, alignability track from UCSC browser showing assembly gaps and low mappability regions.

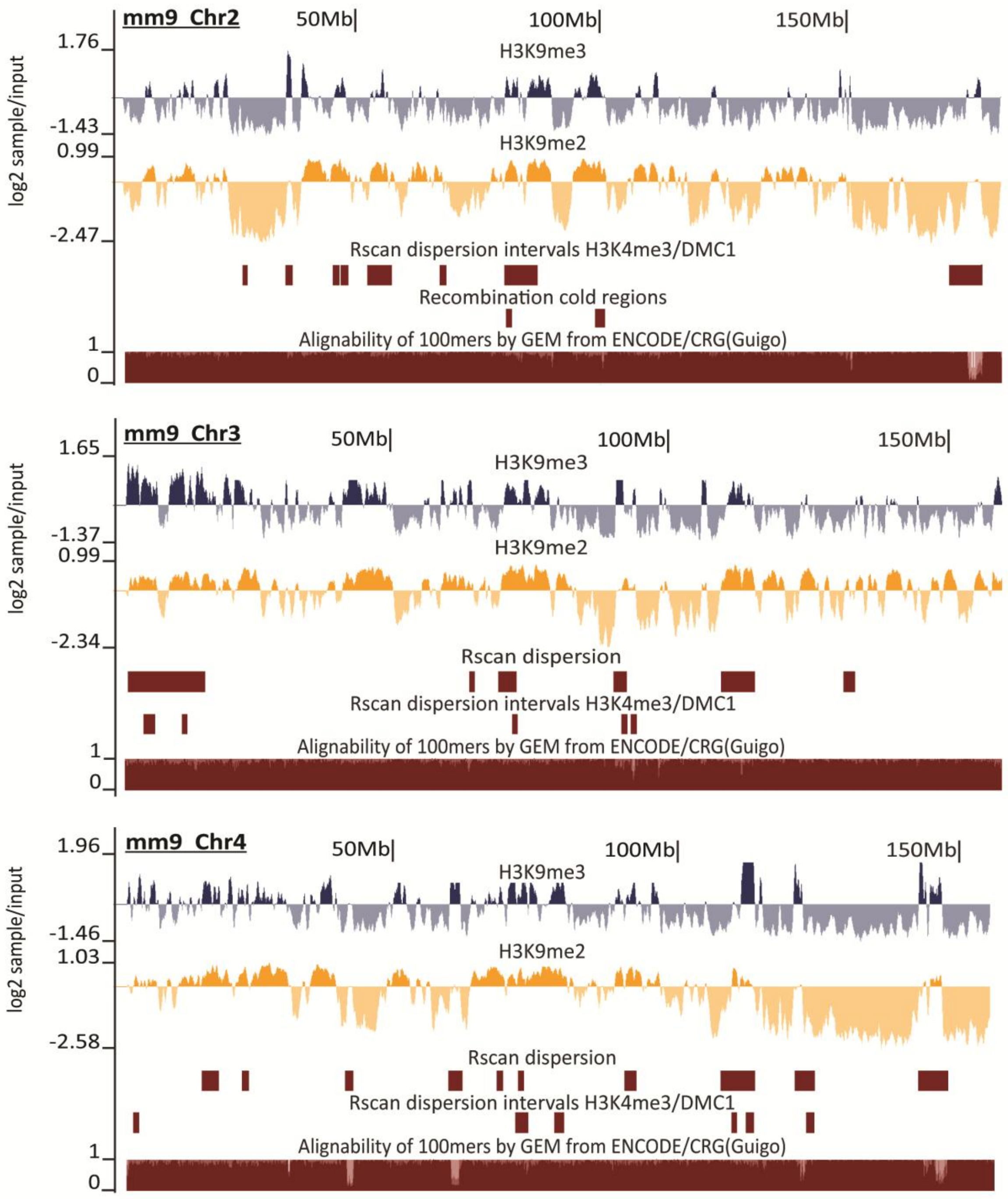
mm9_Chr2
50Mb
100Mb
150Mb
log2 sample/input
1.76
-1.43
0.99
-2.47
H3K9me3
H3K9me2
Rscan dispersion intervals H3K4me3/DMC1
Recombination cold regions
Alignability of 100mers by GEM from ENCODE/CRG(Guigo)
1
0
mm9_Chr3
50Mb
100Mb
150Mb
log2 sample/input
1.65
-1.37
0.99
-2.34
H3K9me3
H3K9me2
Rscan dispersion
Rscan dispersion intervals H3K4me3/DMC1
Alignability of 100mers by GEM from ENCODE/CRG(Guigo)
1
0
mm9_Chr4
50Mb
100Mb
150Mb
log2 sample/input
1.96
-1.46
1.03
-2.58
H3K9me3
H3K9me2
Rscan dispersion
Rscan dispersion intervals H3K4me3/DMC1
Alignability of 100mers by GEM from ENCODE/CRG(Guigo)
1
0

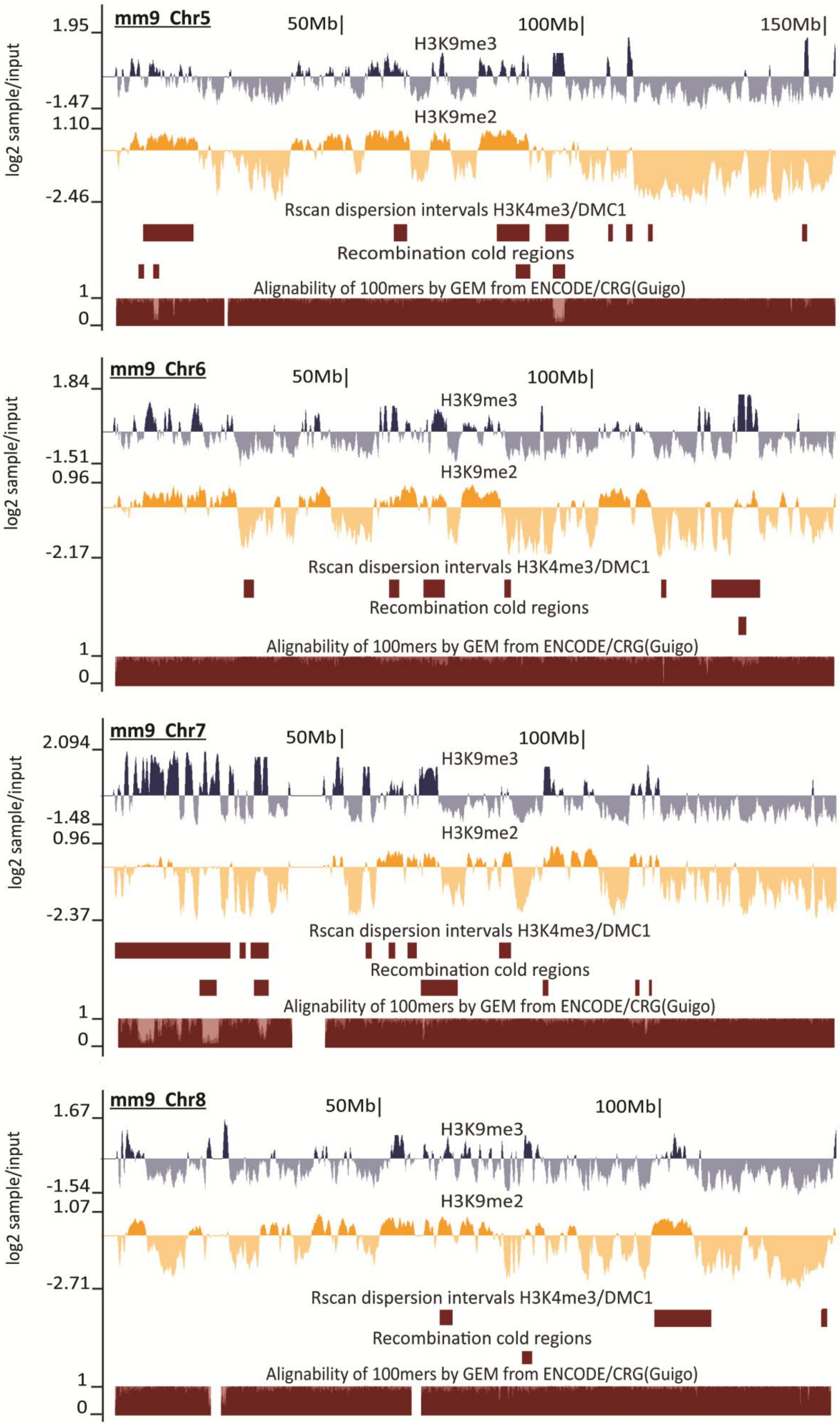

mm9_Chr5
50Mb|
100Mb|
150Mb|
1.95
-1.47
1.10
-2.46
log2 sample/input
H3K9me3
H3K9me2
Rscan dispersion intervals H3K4me3/DMC1
Recombination cold regions
Alignability of 100mers by GEM from ENCODE/CRG(Guigo)
1
0
mm9_Chr6
50Mb|
100Mb|
1.84
-1.51
0.96
-2.17
log2 sample/input
H3K9me3
H3K9me2
Rscan dispersion intervals H3K4me3/DMC1
Recombination cold regions
Alignability of 100mers by GEM from ENCODE/CRG(Guigo)
1
0
mm9_Chr7
50Mb|
100Mb|
2.094
-1.48
0.96
-2.37
log2 sample/input
H3K9me3
H3K9me2
Rscan dispersion intervals H3K4me3/DMC1
Recombination cold regions
Alignability of 100mers by GEM from ENCODE/CRG(Guigo)
1
0
mm9_Chr8
50Mb|
100Mb|
1.67
-1.54
1.07
-2.71
log2 sample/input
H3K9me3
H3K9me2
Rscan dispersion intervals H3K4me3/DMC1
Recombination cold regions
Alignability of 100mers by GEM from ENCODE/CRG(Guigo)
1
0

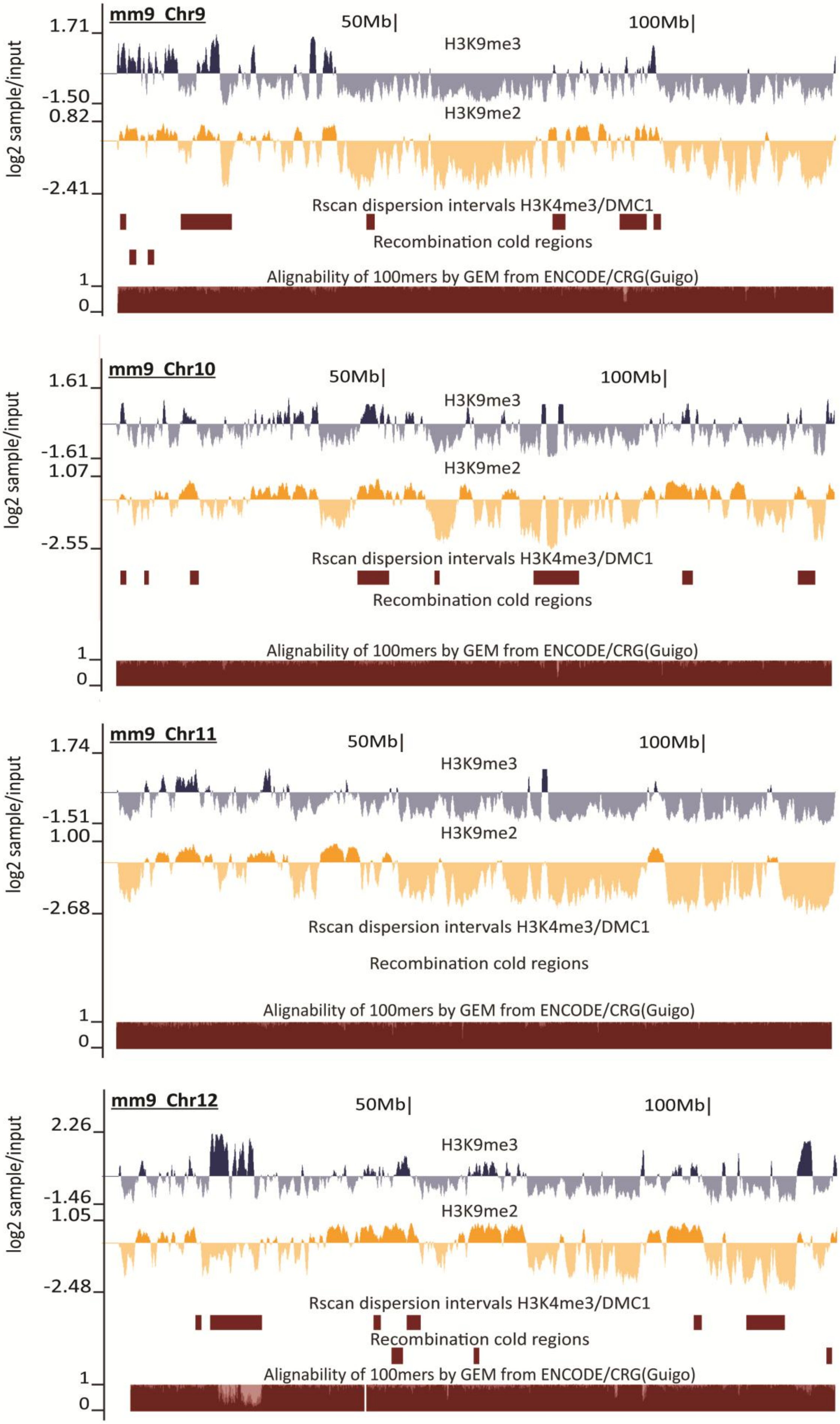
mm9 Chr9
50Mb|
100Mb|
1.71
-1.50
0.82
-2.41
1
0
log2 sample/input
H3K9me3
H3K9me2
Rscan dispersion intervals H3K4me3/DMC1
Recombination cold regions
Alignability of 100mers by GEM from ENCODE/CRG(Guigo)
mm9 Chr10
50Mb|
100Mb|
1.61
-1.61
1.07
-2.55
1
0
log2 sample/input
H3K9me3
H3K9me2
Rscan dispersion intervals H3K4me3/DMC1
Recombination cold regions
Alignability of 100mers by GEM from ENCODE/CRG(Guigo)
mm9 Chr11
50Mb|
100Mb|
1.74
-1.51
1.00
-2.68
1
0
log2 sample/input
H3K9me3
H3K9me2
Rscan dispersion intervals H3K4me3/DMC1
Recombination cold regions
Alignability of 100mers by GEM from ENCODE/CRG(Guigo)
mm9 Chr12
50Mb|
100Mb|
2.26
-1.46
1.05
-2.48
1
0
log2 sample/input
H3K9me3
H3K9me2
Rscan dispersion intervals H3K4me3/DMC1
Recombination cold regions
Alignability of 100mers by GEM from ENCODE/CRG(Guigo)

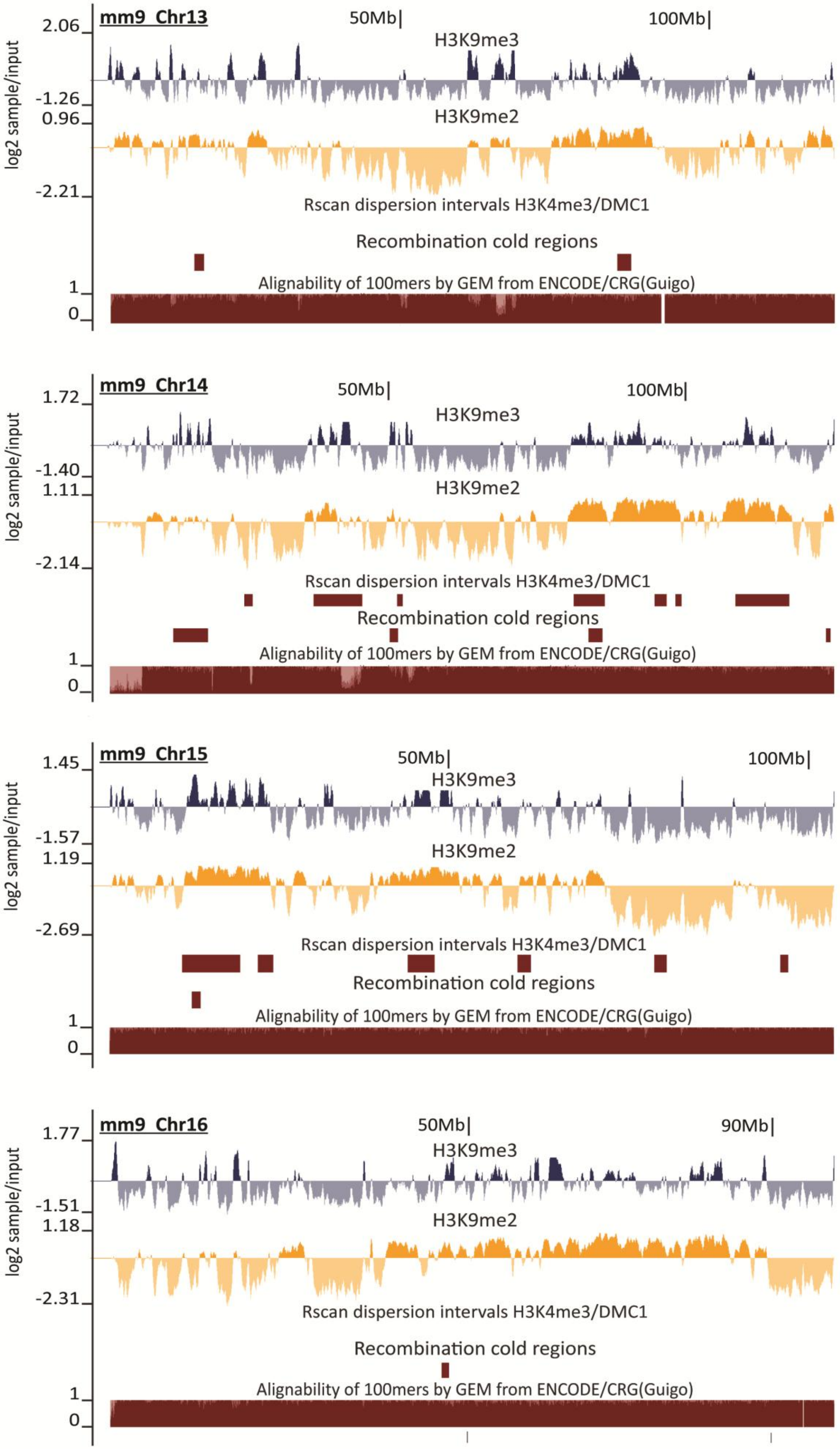
mm9_Chr13
50Mb|
100Mb|
log2 sample/input
2.06
-1.26
0.96
-2.21
H3K9me3
H3K9me2
Rscan dispersion intervals H3K4me3/DMC1
Recombination cold regions
Alignability of 100mers by GEM from ENCODE/CRG(Guigo)
1
0
mm9_Chr14
50Mb|
100Mb|
log2 sample/input
1.72
-1.40
1.11
-2.14
H3K9me3
H3K9me2
Rscan dispersion intervals H3K4me3/DMC1
Recombination cold regions
Alignability of 100mers by GEM from ENCODE/CRG(Guigo)
1
0
mm9_Chr15
50Mb|
100Mb|
log2 sample/input
1.45
-1.57
1.19
-2.69
H3K9me3
H3K9me2
Rscan dispersion intervals H3K4me3/DMC1
Recombination cold regions
Alignability of 100mers by GEM from ENCODE/CRG(Guigo)
1
0
mm9_Chr16
50Mb|
90Mb|
log2 sample/input
1.77
-1.51
1.18
-2.31
H3K9me3
H3K9me2
Rscan dispersion intervals H3K4me3/DMC1
Recombination cold regions
Alignability of 100mers by GEM from ENCODE/CRG(Guigo)
1
0

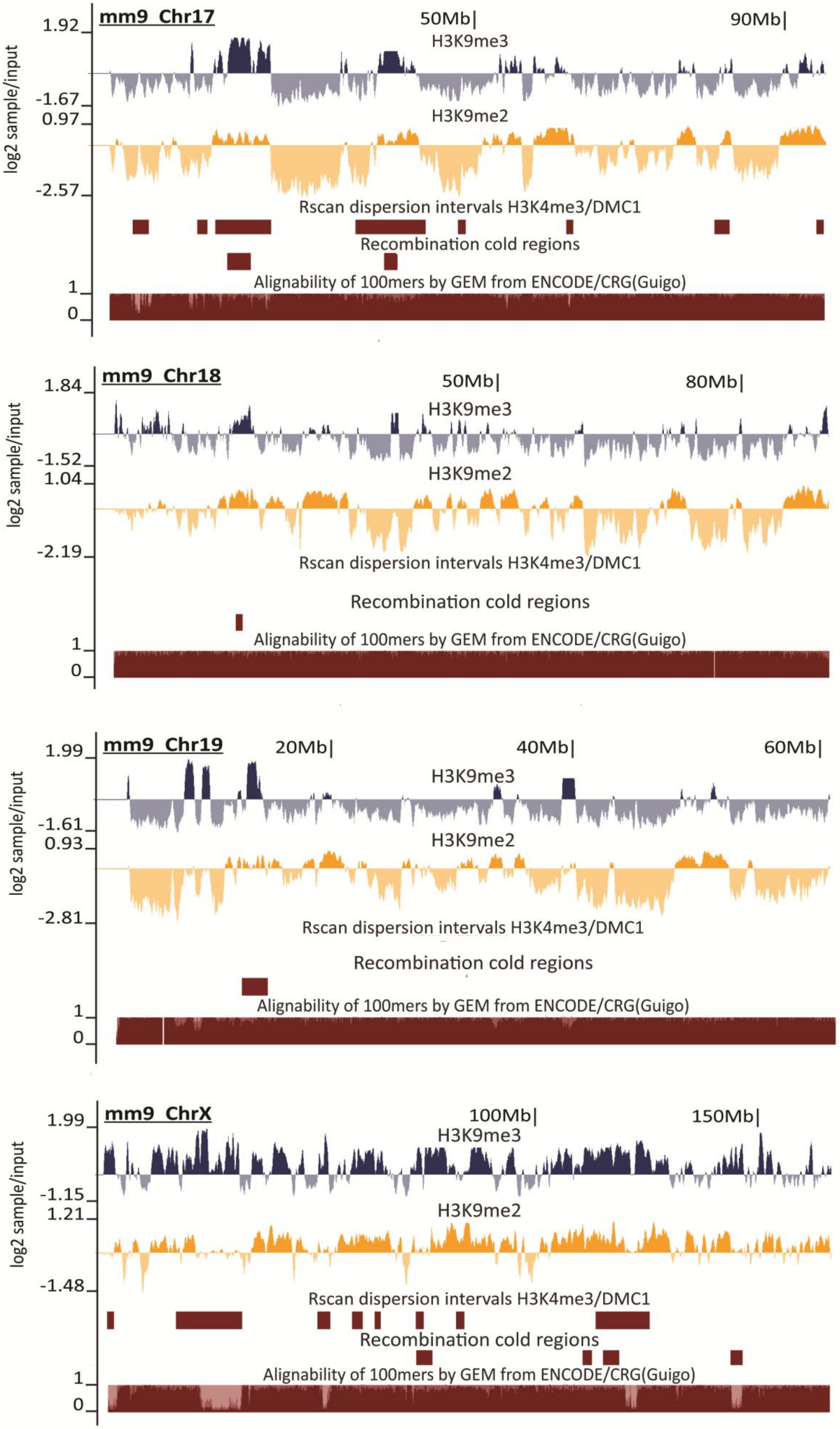
mm9_Chr17
50Mb|
90Mb|
1.92
H3K9me3
-1.67
0.97
H3K9me2
-2.57
Rscan dispersion intervals H3K4me3/DMC1
Recombination cold regions
Alignability of 100mers by GEM from ENCODE/CRG(Guigo)
1
0
log2 sample/input
mm9_Chr18
50Mb|
80Mb|
1.84
H3K9me3
-1.52
1.04
H3K9me2
-2.19
Rscan dispersion intervals H3K4me3/DMC1
Recombination cold regions
Alignability of 100mers by GEM from ENCODE/CRG(Guigo)
1
0
log2 sample/input
mm9_Chr19
20Mb|
40Mb|
60Mb|
1.99
H3K9me3
-1.61
0.93
H3K9me2
-2.81
Rscan dispersion intervals H3K4me3/DMC1
Recombination cold regions
Alignability of 100mers by GEM from ENCODE/CRG(Guigo)
1
0
log2 sample/input
mm9_ChrX
100Mb|
150Mb|
1.99
H3K9me3
-1.15
1.21
H3K9me2
-1.48
Rscan dispersion intervals H3K4me3/DMC1
Recombination cold regions
Alignability of 100mers by GEM from ENCODE/CRG(Guigo)
1
0
log2 sample/input
