# Supplementary material for: Affinity-seq detects genome-wide PRDM9 binding sites and reveals the impact of prior chromatin modifications on mammalian recombination hotspot usage
Source: Epigenetics Chromatin. 2015 Sep 7;8:31. doi: 10.1186/s13072-015-0024-6 (PMC4562113; doi:10.1186/s13072-015-0024-6)

Additional file 7:

Figure S7. Hotspot densities are reduced in gene clusters with silenced expression in spermatocytes. A representative region on mouse Chromosome 3 is shown. The first and second row represent domains enriched (above the line) or depleted (below the line) for H3K9me3 and H3K9me2, respectively; third row – positions of all Affinity-seq sites; fourth row – positions of Affinity-seq sites detected at hotspots *in vivo*; fifth row – positions of genes not expressed in spermatocytes.

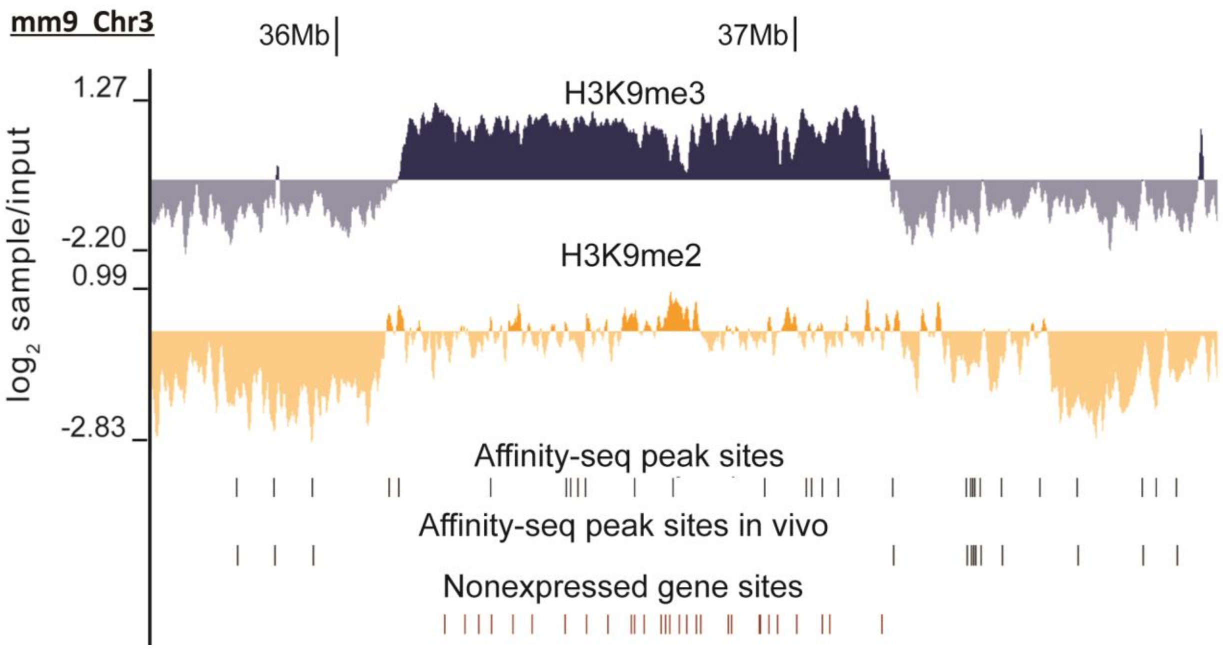

Supplement: Additional file 7: — Figure S7. Hotspot densities are reduced in gene clusters with silenced expression in spermatocytes. [file 13072_2015_24_MOESM7_ESM.pdf]
