# Supplementary material for: Affinity-seq detects genome-wide PRDM9 binding sites and reveals the impact of prior chromatin modifications on mammalian recombination hotspot usage
Source: Epigenetics Chromatin. 2015 Sep 7;8:31. doi: 10.1186/s13072-015-0024-6 (PMC4562113; doi:10.1186/s13072-015-0024-6)

Additional file 8:

Figure S8. Comparison between LOCKs (H3K9me2) in germ cells and LADs in ES cells. A representative region on mouse Chromosome 3 is shown. Top row, H3K9me2 in germ cells; bottom row, LADs in ES cells.

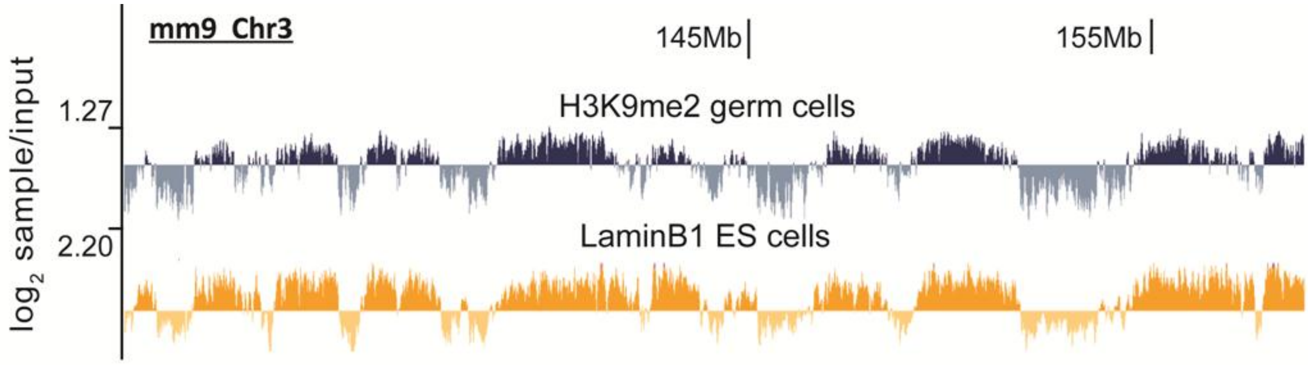

Supplement: Additional file 8: — Figure S8. Comparison between LOCKs (H3K9me2) in germ cells and LADs in ES cells. [file 13072_2015_24_MOESM8_ESM.pdf]
